# Targeting the Urotensin II/UT G Protein-Coupled Receptor to Counteract Angiogenesis and Mesenchymal Hypoxia/Necrosis in Glioblastoma

**DOI:** 10.3389/fcell.2021.652544

**Published:** 2021-04-14

**Authors:** Vadim Le Joncour, Pierre-Olivier Guichet, Kleouforo-Paul Dembélé, Alexandre Mutel, Daniele Campisi, Nicolas Perzo, Laurence Desrues, Romain Modzelewski, Pierre-Olivier Couraud, Jérôme Honnorat, François-Xavier Ferracci, Florent Marguet, Annie Laquerrière, Pierre Vera, Pierre Bohn, Olivier Langlois, Fabrice Morin, Pierrick Gandolfo, Hélène Castel

**Affiliations:** ^1^UNIROUEN, INSERM U1239, DC2N, Institute for Research and Innovation in Biomedicine (IRIB), Normandie Rouen Université, Rouen, France; ^2^EA 4108, Laboratoire d’Informatique, de Traitement de l’Information et des Systèmes (LITIS), University of Rouen, Mont-Saint-Aignan, France; ^3^Université de Paris, Institut Cochin, Inserm U1016, CNRS UMR 8104, Paris, France; ^4^Neuro-Oncology Department, Hospices Civils de Lyon, Hôpital Neurologique, Bron, France; ^5^Institute NeuroMyoGéne, INSERM U1217/CNRS UMR 5310, Lyon, France; ^6^University Claude Bernard Lyon 1, Université de Lyon, Lyon, France; ^7^Neurosurgery Service, Rouen CHU Hospital, Rouen, France; ^8^Anathomocytopathology Service, Rouen CHU Hospital, Rouen, France

**Keywords:** glioblastoma, urotensin II, UT receptor, angiogenesis, necrosis, biased ligand

## Abstract

Glioblastomas (GBMs) are the most common primary brain tumors characterized by strong invasiveness and angiogenesis. GBM cells and microenvironment secrete angiogenic factors and also express chemoattractant G protein-coupled receptors (GPCRs) to their advantage. We investigated the role of the vasoactive peptide urotensin II (UII) and its receptor UT on GBM angiogenesis and tested potential ligand/therapeutic options based on this system. On glioma patient samples, the expression of UII and UT increased with the grade with marked expression in the vascular and peri-necrotic mesenchymal hypoxic areas being correlated with vascular density. *In vitro* human UII stimulated human endothelial HUV-EC-C and hCMEC/D3 cell motility and tubulogenesis. In mouse-transplanted Matrigel sponges, mouse (mUII) and human UII markedly stimulated invasion by macrophages, endothelial, and smooth muscle cells. In U87 GBM xenografts expressing UII and UT in the glial and vascular compartments, UII accelerated tumor development, favored hypoxia and necrosis associated with increased proliferation (Ki67), and induced metalloproteinase (MMP)-2 and -9 expression in Nude mice. UII also promoted a “tortuous” vascular collagen-IV expressing network and integrin expression mainly in the vascular compartment. GBM angiogenesis and integrin αvβ3 were confirmed by *in vivo* ^99m^Tc-RGD tracer imaging and tumoral capture in the non-necrotic area of U87 xenografts in Nude mice. Peptide analogs of UII and UT antagonist were also tested as potential tumor repressor. Urotensin II-related peptide URP inhibited angiogenesis *in vitro* and failed to attract vascular and inflammatory components in Matrigel *in vivo*. Interestingly, the UT antagonist/biased ligand urantide and the non-peptide UT antagonist palosuran prevented UII-induced tubulogenesis *in vitro* and significantly delayed tumor growth *in vivo.* Urantide drastically prevented endogenous and UII-induced GBM angiogenesis, MMP, and integrin activations, associated with GBM tumoral growth. These findings show that UII induces GBM aggressiveness with necrosis and angiogenesis through integrin activation, a mesenchymal behavior that can be targeted by UT biased ligands/antagonists.

## Introduction

Malignant gliomas and mainly glioblastomas (GBMs) are the most common group of primary brain tumors with an incidence of 8.9 cases per 100,000 persons/year in the United States (CBTRUS 2008–2012). According to the WHO classification of 2016, three major diagnostic subtypes of gliomas are individualized in three groups: (i) IDH-mutant, (ii) IDH-wildtype for most comprising GBM, and (iii) IDH not otherwise specified (Louis et al., 2016). GBMs are also characterized by common histopathological features including heterogeneity with regional high abnormal vascularized networks with necrotic foci, surrounded by hyper-cellular areas of “pseudopalisading” cells (Karsy et al., 2012; Alifieris and Trafalis, 2015), correlated with response to treatment (Stupp et al., 2005; Wen and Brandes, 2009). This GBM inter- and intra- heterogeneity was highlighted by the identification of major transcriptomic subgroups including the proneural (PN), neural (N), classical (CL), and mesenchymal (MES) GBM subgroups (Verhaak et al., 2010), potentially recapitulated within a GBM resection fragment (Wick and Kessler, 2018), strongly supporting the heterogeneous and constantly metamorphosing nature of GBM (Aubry et al., 2015).

The pronounced vascularization of GBM exhibits aberrant, malfunctioning, and leaky features resulting in vasogenic edema and increased tissue hypoxia sustaining the increased tumor malignancy (Takano, 2012; Bougnaud et al., 2016; Cavazos and Brenner, 2016). Angiogenic regulators are secreted by GBM cells but also infiltrating myeloid cells such as tumor-associated macrophages (TAMs) and Tie-expressing monocytes via ligands expressed by the tumor and/or stromal cells and targets present at the endothelial level (Eelen et al., 2020). The well-known anti-human VEGFA antibody bevacizumab has been shown not only to reduce tumor edema, angiogenesis, and disease burden but also to provoke adaptive escape mechanisms involving hypoxia by pruning tumor blood vessels, switching to a glycolytic metabolism, neo-vascularization, and/or infiltrative tumor growth (Norden et al., 2008; Eelen et al., 2020). Tumor relapse is associated with cell invasion of brain parenchyma likely involving chemotactic factors (Xie et al., 2014), such as cytokines, growth factors, chemokines, or vasoactive peptides activating G protein-coupled receptors (GPCRs) (Hembruff and Cheng, 2009; Borsig et al., 2014). Glioma cells but also macrophages are a major source of chemoattractants and they express one or more chemoattractant GPCRs to their advantage (Cherry and Stella, 2014; Gagliardi et al., 2014; Zhou et al., 2014), allowing leukocyte attraction or exacerbating angiogenesis (Glass and Synowitz, 2014). Some vasoactive peptides and their GPCRs, conventionally involved in the angiogenic and inflammatory processes, constitute promising therapeutic targets in angiogenic and invasive tumors. The selective blockade of the angiotensin II (Ang-II) receptor AT1 by losartan inhibits the development of murine glioma and decreases tumor neo-angiogenesis (Rivera et al., 2001; Arrieta et al., 2005). The antagonism of endothelin-1 (ET-1) receptor by atrasentan in a phase I clinical study showed partial responses, lasting stabilization, or even absence of progression of GBM (Phuphanich et al., 2008). The immunoneutralization of adrenomedullin (ADM) caused a dramatic inhibition of prostate and GBM tumor development in Nude mice, accompanied by a drastic diminution of intratumoral vascular density (Kaafarani et al., 2009; Metellus et al., 2011).

Human urotensin II (UII) (Coulouarn et al., 1998) and its peptide paralog UII-related peptide (URP) (Sugo et al., 2003) are cyclic neuropeptides of 11 and 8 amino acids in human, respectively, and exhibit a fully conserved C-terminal cyclic hexapeptide (CFWKYC) core that plays a major role in biological activity (Brulé et al., 2014; Table 1). UII and URP are considered the most potent endogenous vasoactive molecules known so far acting through a common GPCR called UT, being evolutionary linked to chemokine GPCRs (Ames et al., 1999; Brulé et al., 2014; Castel et al., 2017). First studies established that the urotensinergic system is involved in migration of rat fibroblasts (Zhang et al., 2008), rat endothelial progenitor cells (Xu et al., 2009), human monocytes (Segain et al., 2007), and *in vitro* angiogenesis (Spinazzi et al., 2006), therefore stressing on the potential chemokine and pro-angiogenic status of UII in cancer. Indeed, UT receptor was shown to regulate migration of prostatic (Grieco et al., 2011), bladder (Franco et al., 2014), and colon (Federico et al., 2014) cancer cell lines. More recently, we and others proposed UII as an original chemokine (Castel et al., 2017; Sun and Liu, 2019), stimulating lung or colorectal cancer cell proliferation and metalloproteinase-9 (MMP-9) activation (Zhou et al., 2012). In the central nervous system (CNS), UT was detected in the vascular compartment (Clavier et al., 2018), in astroglial processes, and in human native astrocytes and GBM cell lines (Castel et al., 2006; Jarry et al., 2010; Desrues et al., 2012). In GBM cells, UII induces chemotactic migration/adhesion *via* G13/Rho/ROCK/actin polymerization and partially the Gi/o/PI3K pathways involving inhibition of the autophagy process (Lecointre et al., 2015; Coly et al., 2016). The chemoattractant activity of the UII-UT system may play multiple roles during glioma invasion or on microenvironmental cells leading to angiogenesis. In the present study, we characterized the expression of UII peptides and their receptor UT in a series of human glioma biopsies compared with normal brain tissue by immunohistochemistry and from transcriptome array analyses of gliomas from The Cancer Genome Atlas (TCGA) database as well as by quantitative-PCR (qPCR) in glioma cell lines. We found marked increased expression of UII and UT in high malignancy gliomas associated with the vascular component in GBM. The native UII and no other urotensin analog stimulates endothelial tube formation *in vitro* and promotes the *in vivo* recruitment of pro-angiogenic cells in matrices’ sponges. In heterotopic U87 GBM cells xenografted in Nude mice, intratumoral injections of UII accelerated proliferation and GBM growth and exacerbated abnormal angiogenesis associated with hypoxia and necrotic features confirmed by *in vivo* ^99m^Tc-RGD tracer imaging and tumoral capture in non-necrotic areas. The UT antagonist/biased ligand urantide and the non-peptide UT antagonist palosuran prevented UII-induced tubulogenesis *in vitro* and significantly delayed tumor growth *in vivo.* Urantide drastically prevented UII-induced GBM angiogenesis, MMP-2 and MMP-9 expression, and integrin activation associated with GBM growth. The specific blockade of UT receptor signaling should constitute a new multicellular targeting option in the therapeutic arsenal against GBM.

**TABLE 1:** Names, sequences, and characteristics of the different urotensinergic peptide and non-peptide ligands used in the study.

**UT ligand**	**Known function**	**Sequence**
Human UII (UII)	Endogenous agonist	HTPD[CFWKYC]V
Murine UII (mUII)		pEHGAAPE[CFWLYC]I
UII-related peptide (URP)		HA[CFWKYC]V
UII_4__–__11_	Synthetic agonist	HD[CFWKYC]V
Urantide	Peptide antagonist/biased ligand	D[*Pen*-F_D_W-*Orn*-YC]V
Palosuran	Non-peptide antagonist	1-[2-(4-benzyl-4-hydroxy-piperidin-1-yl)-ethyl]-3-(2-methyl-quinolin-4-yl)-urea sulfate salt)

*Cyclic core domain (disulfide bonds between two C residues) is represented by brackets. Unnatural amino acids: Orn, ornithine; Pen, penicillamine.*

## Materials and Methods

### Tumor Patient Samples

Non-tumoral brain tissues and brain glioma tumors were obtained from patients cared at the Rouen CHU Hospital in France from 2008 to 2014. Sixty-six tumors were taken from the Center of Biological Resources located in the Department of Pathology, Rouen University Hospital. The selection criteria were a diagnosis of glial tumor, surgical resection or biopsy, treated by surgery alone, surgery plus radiotherapy, surgery plus radiotherapy and Temozolomide chemotherapy, or surgery plus radiotherapy and PCV (Procarbazine, Lomustine, and Vincristine) chemotherapy (Stupp protocol), and patients with completed clinical information (see Table 2 for detailed information). All patients provided written informed consent for the study. The age of patients, their clinical outcomes, and tumor histopathologic classifications were typical of the category of adult with diffuse glioma (Verhaak et al., 2010; Brennan et al., 2013).

**TABLE 2:** Clinico-demographical parameters of the different glioma groups studied retrospectively and prospectively.

**CNS tissues**	**Grade WHO 2016**	**Grade WHO 2008**	**Subtype**	***N***	**Median age**	**Gender (M/F)**	**Localization**	**Treatment**
**Gliomas**	**I**		Pilocytic astrocytoma	**8**	16 (2–25)	4/4	CH (*n* = 6), V (*n* = 2)	CR
	**II**			**13**	36 (26–60)	8/5	SC (*n* = 11), BG (*n* = 1), BS (*n* = 1)	CR alone (*n* = 4), T (*n* = 5), RT (*n* = 4)
		AII	Diffuse astrocytoma, IDH1-mutant	3				
		AII	Diffuse astrocytoma, IDH1-wildtype	3				
		OII	Oligodendroglioma, IDH1-mutant and 1p/19q-codeleted	3				
		OIII	Oligodendroglioma, IDH1-wildtype and 1p/19q-codeleted	1				
		OAII	Oligoastrocytoma, NOS	3				
	**III**			**15**	52 (24–71)	11/4	SC (*n* = 15)	CR alone (*n* = 1), STUPP (*n* = 6), RT + PCV (*n* = 1), RT alone (*n* = 6), T alone (*n* = 1)
		AIII	Anaplastic astrocytoma, IDH1-mutant	1				
		AIII	Anaplastic astrocytoma, IDH1-wildtype	6				
		OIII	Anaplastic oligodendroglioma, IDH1-mutant and 1p/19q-codeleted	4				
		OAIII	Anaplastic oligoastrocytoma, NOS	4				
	**IV**			**24**	56 (23–75)	16/8	SC (*n* = 23), M (*n* = 1)	STUPP (*n* = 23), RT alone (*n* = 1)
		GBM	Glioblastoma, IDH1-mutant	5				
		GBM	Glioblastoma, IDH1-wildtype	19				
**Non-tumoral**				**6**	45 (32–49)	1/4	_	_
			Hippocampal sclerosis	4				
			Focal cortical dysplasia	1				
			Normal cerebellum	1				

**FIGURE 1:**
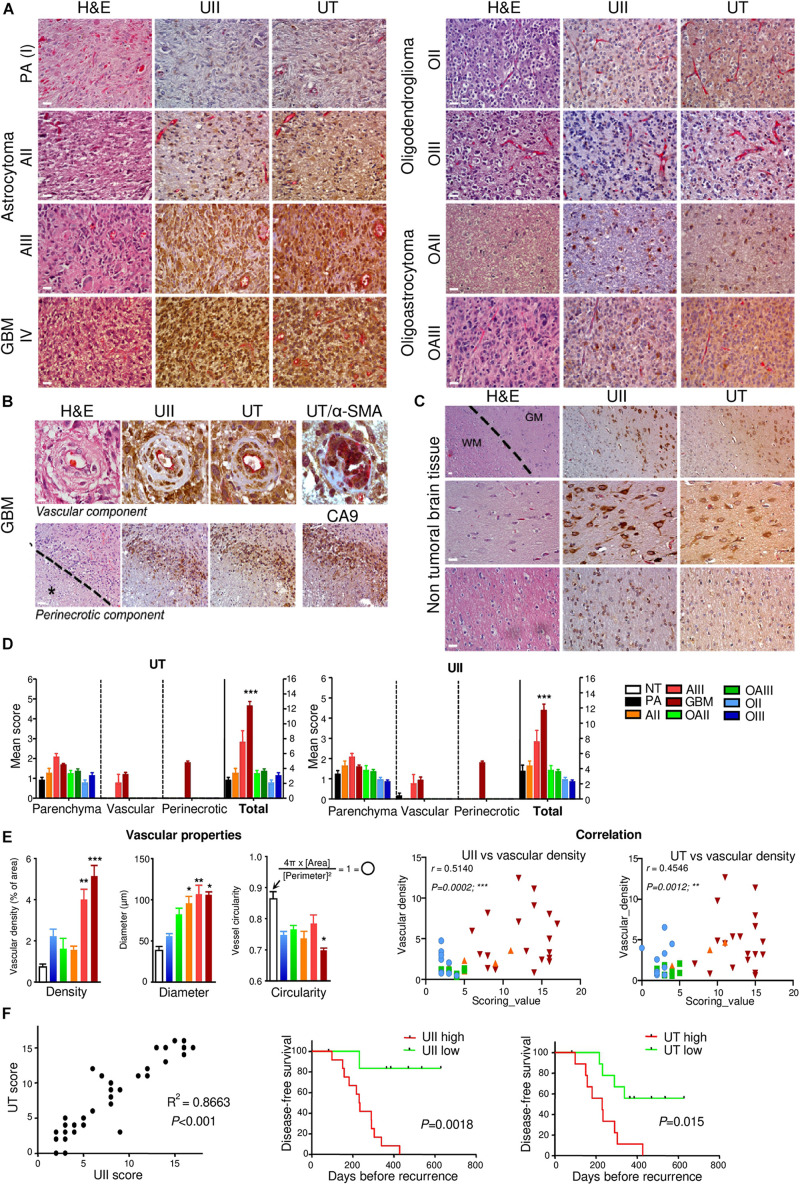
Characterization of UII and UT expression in non-tumoral and glioma samples. **(A)** UII and UT co-labeling (brown) with anti-CD34 immunoreactivity (pink) on consecutive sections from different grades of astrocytomas [PA (I), AII, and AIII], oligodendrogliomas (OII and OIII), and oligoastrocytomas (OAII and OAIII) along the histopathological classification showed a more intense staining in AIII and GBM tumors (left panels) compared with O and mixed OA tumors (right panels). **(B)** UII and UT immunohistochemical co-expression with anti-CD34 structures on consecutive sections of GBM samples revealed staining in vascular cells of hyperplasic vessels (upper panels) and in pseudopalisading cells around areas of necrosis (lower panels). Co-expression of UT (brown) with α-SMA (pink) (upper right panel) in smooth muscle/pericyte vascular components. CA9 staining (brown) highlights hypoxic environment around necrotic areas (lower right panel) exhibiting immunoreactivity for UII and UT. The black star and the dotted line in the lower panels indicate necrosis location. **(C)** UII and UT expression on consecutive sections of three different non-tumoral tissues showed a strong staining in the gray matter (GM) especially in neurons (upper and middle panels) compared to oligodendrocytes from the GM (peri-neuronal satellitosis) and in the white matter (WM) (upper and lower panels). The dotted line delineates the frontier between GM and WM. **(D)** Quantification of experiments presented in **(A,B)** and represented as the mean ± SEM of UT (left panel) and UII (right panel) scores (see section “Materials and Methods”). *P < 0.05; **P < 0.01; ***P < 0.001 (one-way ANOVA and multiple comparison test with Tukey’s correction). **(E)** Vascular density, diameter, and circularity quantified from NT and malignant glioma (AII/OII to GBM) samples based on the CD34-positive structures and histological characteristics from, at least in part, three different images per patient tumor slice. *Left*, Histograms of mean ± SEM (Kruskal–Wallis and Dunn’s post-test: ***P < 0.001). *Right*, Scatter plot of the correlation between UII scoring value versus vascular density (left) and UT scoring value versus vascular density (right). Score correlation in glioma showed a significant correlation between UII and UT and vascular density more particular within AIII and GBM samples. **(F)** Total score correlation in glioma samples showed a significant correlation between UII and its receptor UT. Disease-free survival curves of 20 primary GBM for UII (left panel) and UT (right panel). In **(E,F)**, the correlation is considered significant when *P* < 0.05, *r* represents the Pearson correlation coefficient. H/E: Hematoxylin–Eosin staining. Scale bar = 10 μm for **(A,B)**, and (**C**, middle and lower panels), and 20 μm for (**C**, upper panel).

*Gradings are indicated along histopathological classification (see Figure 1) and according to the WHO classification 2016 from last information available in 2017. BG, basal ganglia; BS, brainstem; CH, cerebellar hemisphere; CR, chirurgical resection; IDH1mut, IDH1(R132H) positive (immunochemistry); IDH1wt, IDH1(R132H) negative (immunochemistry); M, medullary; N, number of samples; NOS, not otherwise specified; PCV, procarbazine, lomustine (CCNU) and vincristine; RT, radiotherapy; SC, supratentorial cortex; STUPP, therapy protocol involving radiotherapy plus concomitant and adjuvant Temozolomide; T, Temozolomide; V, vermis. Bold values correspond to the total of samples per/grade in the table.*

### Immunohistochemistry

Four-micrometer sections were obtained from formalin-fixed paraffin-embedded tissues. Before antibody staining, heat-induced antigen retrieval was performed in a sodium citrate buffer solution at 95°C for 45 min. Immunohistochemistry was performed using Envision GI2 Doublestain System Rabbit/Mouse kit (Dako, K5361) according to the manufacturer’s protocol. Primary antibodies against Urotensin II (Sigma-Aldrich, HPA017000, 1:50), urotensin II receptor (Santa Cruz, sc20940, 1:50), CD34 (Novocastra, RTU-END, 1:1), CA9 (Abcam, ab15086, 1:1000), and α-SMA (Sigma-Aldrich, A2547, 1:500) were used and incubated overnight at 4°C. Rabbit polyclonal and mouse monoclonal antibodies were revealed with DAB and Permanent red, respectively. Nuclei were counterstained with hematoxylin. For each tumor series, a consecutive section was stained using hematoxylin–eosin (H&E) for structural information.

### Scoring of UII/UT Staining in Human Biopsies

Tissue samples were independently scored by two pathologists (AL and FM) and one scientist (POG). The percentage of positive cells (P) was determined as follows: *P* = 0, no positive cells; *P* = 1, <10% of positive cells; *P* = 2, 11–50% of positive cells, *P* = 3, >50% of positive cells. Semi-quantitative evaluation of immunolabeling intensity (I) was determined as follows: *I* = 0, no staining; *I* = 1, weak expression; *I* = 2, moderate expression; *I* = 3, strong expression. The score was expressed as the sum of (P + I) comprised between 0 and 6. For each sample, we scored the expression of UII and UT in three different tumor locations: parenchyma, vascular, and perinecrotic components. The total score was the sum of each score.

### Culture of Cell Lines

The human glioma SW1088 and U87 cell lines were obtained from ATCC (LGC Standards, Molsheim, France) and 8MG, 42MG, and U251 cell lines were provided by Pr. J. Honnorat (CRNL, Lyon, France). Culture media components were purchased from Life Technologies (Thermo Fisher Scientific, Saint-Aubin, France) or Lonza (Levallois-Perret, France). U87 cells were maintained in minimum essential medium (DMEM) supplemented with sodium pyruvate (1%), non-essential amino acids (1%), and antibiotics (1%) all from Fischer Scientific (Illkirch, France) and heat-inactivated fetal bovine serum (FBS, 10%). Human umbilical vein (HUV-EC-C, from ATCC) and human cerebral microvascular (hCMEC/D3) (Weksler et al., 2005, 2013) endothelial cell lines were maintained in endothelial basal medium-2 (Lonza, Bâle, Switzerland) containing chemically defined concentrated lipids (1%, Fischer Scientific), HEPES (1%, Fischer Scientific), hydroxycortisone (0.2 μg/ml, Sigma-Aldrich), bFGF-2 (50 ng/ml, Eurobio Abcys, Courtaboeuf Les Ulis, France), ascorbic acid (1 μg/ml, Fischer Scientific), and gold-defined FBS (5%, PAA Laboratories). When cultures reached 90% of confluence, cells were harvested and prepared for *in vitro* experiments according to the following procedures or were just rinsed in PBS for transplantation in Nude mice.

### Vascular Characteristic of Malignant Glioma Patient Samples

For each patient sampled tumor, at least three microphotographs were taken (Nikon 1 widefield microscope) from slices stained with an anti-CD34, and saved as 2560 × 1920 pixels pictures. Three different vascular properties were analyzed on vascular CD34+ structures with vascular histological characteristics by using ImageJ software (NIH, Bethesda, MD, United States). First, the vascular density was quantified by manually isolating each microvessel in the field and summed, and then microvessel coverage area was normalized to the whole tumoral area. Vessel diameter was quantified from transversal vessel sections, after manual selections, and the highest radius value was used for the quantification by using ImageJ shape descriptors feature. Vessel circularity was quantified on transversal sections to estimate vessel irregularities, after manual selections, and measured by using the following equation:

$$\frac{4\,\pi \,\left[A\,r\,e\,a\right]}{{\left[P\,e\,r\,i\,m\,e\,t\,e\,r\right]}^{2}}$$

### Quantitative Real-Time PCR Analysis

Total RNA was isolated from tumor cell line extracts using TRIzol reagent according to the manufacturer’s protocol (Sigma-Aldrich, Saint-Quentin-Fallavier, France). First-strand cDNA were transcribed from 1 μg of total RNA in quadruplicates using the Superscript II reverse transcriptase (random primers method, Promega, Charbonnières-les-bains, France). For quantitative RT-PCR, cDNA amplification was monitored using SYBR Green (Thermo Fisher Scientific, Illkirch, France) chemistry on the real-time PCR system (QuantStudio3, Applied Biosystems, Zug, Switzerland). Primers were designed by retrieving nucleotide sequence from NCBI gene database and using the Primer3Plus program^1^. The specific primers targeting UTS2 (encoding UII), UTS2D (encoding URP), UTS2R (encoding UT), and MMP9 and ITGAV (encoding integrin αv) mRNA were synthesized by Eurofins genomics (Les Ulis, France), and their sequences are listed in Supplementary Table 1. The conditions for PCR reactions were 45 cycles, 95°C/1 s, 60°C/20 s, a step specific for UII mRNA (73°C/5 s), and 95°C/1 s using the primers specified in Supplementary Table 1. Minus-reverse transcription (“-RT”) controls were systematically performed, and the quality of PCR products was evaluated by generating a melting curve, which was also used to verify the absence of PCR artifacts (primer dimers) or non-specific PCR products. Samples were amplified in triplicates (three different culture flasks and three different RT) and relative mRNA copy levels were determined using the comparative ΔΔCt method. Glyceraldehyde-3-phosphate dehydrogenase (GAPDH) or ubiquitin C (UBC) transcript levels were used as a reference to control mRNA levels and stability within each cell line. Results were analyzed by using the Quantstudio design and analysis software (Applied Biosystems) and are expressed as mean of gene of interest expression relative to GAPDH or UBC reference gene.

### Tumor Data Base Analysis

The clinical and molecular data on low-grade glioma and GBM samples for this study were downloaded from The Cancer Genome Atlas database^2^ firstly in 2014, and the molecular information was again downloaded in February 2016. The clinical information used was the overall patient survival. The molecular data used were mRNA expression levels of UTS2, UTS2D, and UTS2R obtained from RNA-seq information by means of Illumina TruSeq Kit Paired-end Sequencing on Illumina HiSeq2000 (Cancer Genome Atlas Research Network, 2008), and the gene expression level within the GBM subgroups of the Verhaak classification was obtained from different microarray platforms previously processed and summarized (Verhaak et al., 2010; Brennan et al., 2013). Detection of somatic variants (IDHmut) from the TCGA Whole-Exome Sequencing and RNA-Seq data was done using RADIA. RADIA is a computational method combining the patient-matched normal and tumor DNA with the tumor RNA (Lecointre et al., 2015; Coly et al., 2016). The mRNA levels were represented as “normalized count” corresponding to a transformation of the “raw_count.” For each gene of interest, all “raw_count” values were divided by the 75th percentile of the column patient (after removing zeros) and multiplied by 1,000. For the gene-level survival analysis, we divided the corresponding cohorts (all glioma) by the mean gene expression level (low or high) of UTS2, UTS2D, or UTS2R. We then used a log rank (Mantel–Cox) test to compare the survival durations between the two groups.

### Western Blot of Pre-Pro UII and UT

Glioblastoma cell lines and hCMEC/D3 cells were treated during 24 h in the absence of FBS and in the absence or the presence of UII (10^–9^ M). Cell lysates (20 μg total proteins) were prepared in ice-cold Lysis Buffer (25 mM Tris–HCl, pH 7.6, 150 mM NaCl, 1% NP40, 1% sodium deoxycholate, and 0.1% SDS), mixed with Laemmli buffer and loaded onto 4–12% polyacrylamide gels (CliniSciences, Nanterre, France). Proteins were transferred onto PVDF membranes, blocked with 5% non-fat milk and 5% bovine serum albumin, and incubated with the anti-pre-pro UII (Sigma, HPA-01700), UT (H-90) (Santa-Cruz, sc-20940), or β-tubulin (Santa-Cruz, sc-9104) overnight at 4°C and then with horseradish peroxidase-conjugated secondary antibodies (Santa-Cruz) for 2 h at room temperature. Immunoreactive bands were visualized by using the ECL Western blotting substrate (GE Healthcare, Aulnay-sous-Bois, France) and their molecular weight were determined by using PageRuler Plus prestained protein ladder (10–250 kDa) markers (Fisher Scientific).

### Cell Migration Assay

Transwell membranes (24 wells, Corning, Fisher Scientific) with 8-μm pores were coated with 25 μg/ml collagen I (Invitrogen) (4°C, 12 h). Endothelial and glioma cells (5 × 10^4^) suspended in 100 μl of culture medium (with 1% gold-defined FBS for endothelial cells) were added onto the upper chamber of the Transwell and UT ligands (600 μl) were added in the bottom chamber. After 24-h incubation at 37°C, cells were rinsed with PBS and the non-migrating cells were removed using a cotton swab from the upper chamber. Migrating cells were fixed in successive methanol baths and stained with H&E, and then membranes were mounted on glass slides with Mowiol (Calbiochem, Molsheim, France). Random phase contrast images were acquired using a digital camera (Nikon D-600), and image analysis was carried out using *ImageJ*’s Cell Counter plugin (NIH, Bethesda, United States).

### *In vitro* Tubulogenesis Assay

Growth factor-reduced Matrigel (Beckon Dickinson, Le Pont-de-Claix, France) was thawed on ice overnight and spread homogeneously (100 μl) in 24-well plates for 30 min at 37°C to allow Matrigel polymerization. Endothelial cells (5 × 10^4^ cells/cm^2^) were seeded in basal medium and incubated for 24 h in the absence or the presence of UT agonists (Phoenix Pharmaceuticals, Inc) (UII, mUII, URP, UII_4__–__11_, Table 1) at 10^–12^ to 10^–7^ M, the UT antagonist/biased ligand urantide (Peptides International, Louisville, KY, United States, Table 1), or the UT antagonist palosuran (Actelion Pharmaceuticals, Allschwil, Switzerland, Table 1) from 10^–10^ to 10^–7^ M. Phase contrast image analysis was carried out using *ImageJ* software.

### Animal Studies

All procedures were performed in accordance with the French Ethical Committee as well as the guidelines of European Parliament directive 2010/63/EU and the Council for the Protection of Animals Used for Scientific Purposes. This project was approved by the “Comité d’Ethique NOrmandie en Matière d’EXpérimentation Animale” CENOMEXA under the National Committee on Animal Experimentation and received the following number N/13-11-12/36/11-17. Animal manipulations were carried out under the supervision of an authorized investigator (H. Castel; authorization no. 76.98 from the Ministère de l’Alimentation, de l’Agriculture et de la Pêche and surgery agreement). For the tumorigenesis studies, 6-week-old male athymic *Swiss* (*nu/nu*) mice (Charles River Laboratories, L’Arbresle, France) were used. For the *in vivo* chemoattraction assay, 3- to 6-week-old male C57Bl/6 mice (Janvier Labs, Saint-Berthevin, France) were used. Mice were housed in sterile cages, in a temperature-controlled room with a 12-h light/12-h dark schedule and fed with autoclaved food and water *ad libitum.*

### *In vivo* Chemoattraction Assay

Male C57B/l6 mice were injected subcutaneously above the pelvis area with 200 μl of liquid growth factor-reduced Matrigel combined either with EG-VEGF (Miltenyi Biotechnologies, Paris, France) or with human recombinant EGF (R&D Systems, Metz, France) (500 ng/ml, each), UT agonists (UII, mUII, URP, and UII_4__–__11_) at 50 ng/ml, UT antagonists/biased ligand palosuran or urantide at 1 μg/ml, or alone as a negative control. Subcutaneous plug incubation continued for 21 days or until mice had to be sacrificed following institutional ethical guidelines criteria. Matrigel plugs were resected and immediately frozen in a −40°C isopentane solution until immunohistochemistry.

### Xenograft Studies

U87 cells were injected subcutaneously into the right flank (3 × 10^7^) of 6-week-old male athymic mice. Mice were randomized into treatment groups after tumors were developed (mean s.c. volume = 100 mm^3^, 400–600 mm^3^). UT agonists (UII, mUII, URP, and UII_4__–__11_; 2.9 ng/kg, each) or UT antagonists/biased ligand (palosuran, 29 ng/kg; urantide 290 ng/kg) or saline solution (vehicle) was administered intratumorally every day. Subcutaneous tumor volumes were measured with a caliper every 2 days, by using the following formula: width × length × height × 0.52. Treatment continued until mice had to be sacrificed (i.e., 2-cm^3^ maximal tumor dimension, cutaneous ulceration, or symptoms) or study end. In some experiments, 30 min before euthanasia, pimonidazole (60 mg/kg, Hypoxyprobe^TM^-1 Omni Kit, Burlington, United States) was administered intravenously, and glioma tissues were collected and immediately frozen in a −40°C isopentane solution until immunohistochemistry.

### Animal Immunohistochemistry

Cryostat sections of Matrigel plugs (10 μm) were processed in a Leica CM1900 cryostat, or xenograft sections (9 μm) were processed in a Leica CM1950 cryostat, mounted directly on slides, and then fixed in 4% paraformaldehyde. Non-specific binding sites were blocked with normal serum (10%) from the animal source of the appropriate corresponding secondary antibody. Immunolabeling was conducted using primary antibodies against UT receptor, F4/80 (Santa Cruz Biotechnologies, Heidelberg, Germany), collagen-IV (Coll-IV, Merck-Millipore, Molsheim, France), α-smooth muscle cell actin (α-SMA) and Prestige-Graded UII (Sigma-Aldrich, Saint-Quentin Fallavier, France), or α_V_ integrin, MMP-2 and MMP-9, and CD31 and CD34 (Abcam, Paris, France), diluted according to the manufacturer’s technical specifications in the incubation media [0.1 M Tris (pH 7.5), 0.15 M NaCl, 10% normal serum, and 0.03% Triton X-100] and then incubated overnight at 4°C. Signal amplification utilized fluorochrome (Alexa 488 or Alexa 594)-conjugated secondary antibodies (Life Technologies, Saint Aubin, France), and cell nuclei DNA was stained with 4’,6-diamidino-2-phenylindole (DAPI, Sigma-Aldrich). Control sections were incubated in the absence of the primary antibodies.

### Micro-SPECT Imaging and ^99m^Tc RGD Radioligand

Cyclo(RGD_D_Y[K-HYNIC]) (HYNIC-RGD) was synthesized on a solid support using standard solid-phase synthesis and was purchased from the PolyPeptide Group (Strasbourg, France). The labeling method following the previously described protocol (Becker et al., 2015) is more detailed in the Supplementary Material, and chemical structures are shown in Supplementary Figure 6A. Other reagents were purchased from Sigma-Aldrich (Saint-Quentin-Fallavier, France). Na^99m^TcO_4_ was obtained by elution, with 0.9% saline, from a commercial ^99^Mo/^99m^Tc generator (Ultra-TechneKow; Covidien, Petten, Netherlands). The RGD-derived peptide purity was greater than 95% as analyzed by reverse-phase high-performance liquid chromatography and mass spectroscopy, and the net peptide content was estimated by elemental analysis.

SPECT imaging was performed using a small animal imaging system with parallel collimators and a 140 keV ± 10% photopeak energy window (Triumph SPECT/CT; GMI, Los Angeles, CA, United States) and GE Healthcare (Fairfield, CT, United States). U87-xenografted Nude mice were injected through the tail vein with ∼30 MBq (in about 0.1 ml) of [^99m^Tc]-HYNIC-RGD under continuous isoflurane anesthesia (2.5% in O_2_, 1 L/min). [^99m^Tc]-HYNIC-RGD dynamic imaging was carried out 1 h after injection and planar images were recorded every minute. [^99m^Tc]-HYNIC-RGD SPECT imaging was carried out 2 h after injection. Treatment of the dynamic imaging is detailed in Supplementary Methods.

### *Ex vivo* Micro-SPECT Imaging of Tumors

In tumor samples examined *ex vivo*, 30 MBq was precisely measured and diluted by a factor of 1000 to obtain a dose calibrator. Background counts were subtracted, and the radioactive decay was corrected to the time of injection. The radioactivity concentration that had accumulated in the tissue samples over the 2-h period following the [^99m^Tc]HYNIC-RGD injection was expressed as a percentage of the injected dose (%ID) and normalized per gram of tissue (%ID/g). For *ex vivo* biodistribution, samples of various tissues were collected 120 min after injection and weighed, and radioactivity was measured using an automatic gamma counter (1470–001 Wizard Gamma Counter; PerkinElmer and Wallac, Turku, Finland) calibrated with a gamma peak calibrator (^129^I). A ^99m^Tc sample was measured using an activity calibrator (MEDI404; Medisystem, Guyancourt, France). Detailed methods of visualization and quantification of radioactivity were provided in the Supplementary Methods.

### Statistical Analyses

Data were expressed as mean ± SEM and GraphPad Prism (version 5; GraphPad Software, Inc., La Jolla, United States) was used for statistical analyses. Student *t-*test was used for parametric comparisons between paired variables, the Mann–Whitney *U* test was used for non-parametric pairwise comparisons, multivariate analyses were done with ANOVA with *post hoc* Dunnett (*in vitro* analyses) or Bonferroni tests (e.g., *in vivo* studies) as appropriate, the vascular property multivariate analyses were done with Kruskal–Wallis and Dunn’s post-test, and survival curves were generated by the Kaplan–Meier method. Correlation is considered significant when *P* < 0.05, and *r* represents the Pearson correlation coefficient. All reported *P*-values were two-sided and considered to be statistically significant at *P* < 0.05.

## Results

### UII and UT Expression in Patient Gliomas and Human Glioma Cell Lines

**FIGURE 2:**
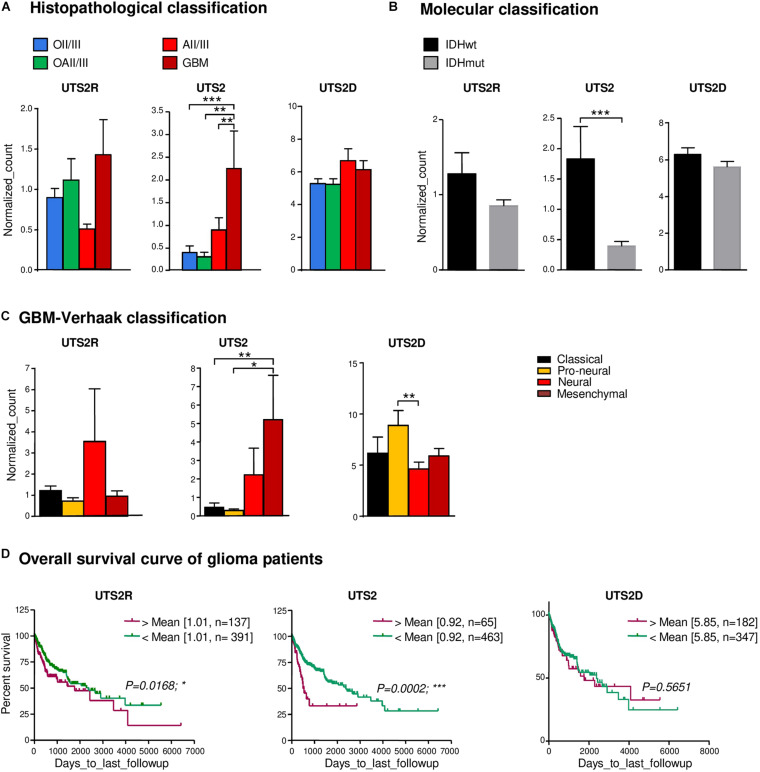
Gene expression of UTS2, UTS2R, and UTS2D and patient survival in gliomas. **(A)** UTS2R, UTS2, and UTS2D are expressed in all histological OII/III, OAII/III, AII/III, and GBM classes and according to the glioma tumor grade, *n* = 66 tumors of the TCGA database. Histograms show gene expression from RNA-seq analysis. The data are from the following cohorts: OII/III, OAII/III, AII/III, and GBM. Of all glioma tissues, UTS2 expression exhibits significant higher levels in GBM compared to others (*P* < 0.0001). **(B,C)** Based on mRNA information on the subgroup classification of GBM from Verhaak et al. (2010) available in TCGA, neural, mesenchymal, and pro-neural groups expressed higher levels of UTS2R, UTS2, and UTS2D, respectively. **(D)** Kaplan–Meier statistical analysis of the prognostic significance of UTS2R, UTS2, and UTS2D. The overall survival period of glioma patients with UTS2R and UTS2 high expression was shorter than that of GBM patients with low expression. Green line, low expression; red line, high expression. *N* indicates the number of samples in each low or high expression group. *P < 0.05; **P < 0.01; ***P < 0.001.

**FIGURE 3:**
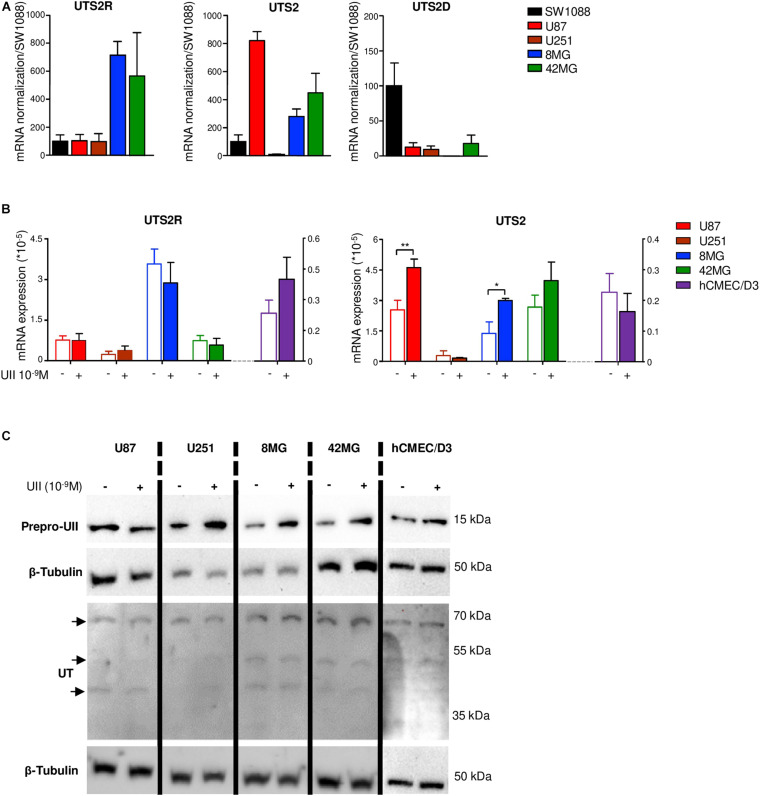
Gene expression of UTS2, UTS2R, and UTS2D in glioma and hCMEC/D3 cell lines. **(A)** UTS2R, UTS2, and UTS2D gene expression were analyzed by qPCR in gliomas. UTS2R, UTS2, and UTS2D mRNA expressions are presented as ΔΔCt, related to the GAPDH gene expression in the anaplastic astrocytoma cell line SW1088 and in U87, U251, 8MG, and 42MG GBM cell lines. Data were expressed as mean ± SEM of three independent cultures and normalized to mRNA first to the housekeeping gene GAPDH and then to levels of gene of interest in SW1088. **(B)** Effect of UII on UTS2 and UTS2R gene expression in U87, U251, 8MG, and 42MG GBM cell lines and the endothelial hCMEC/D3 cell line. Cells were treated by UII (10^–9^ M, 24 h) in the absence of FBS. UTS2R and UTS2 mRNA expressions were expressed as mean ± SEM of ΔΔCt from three independent cultures normalized to the housekeeping gene UBC. Statistical significance of treatments vs. control condition was assessed with Mann–Whitney test. *P < 0.05; **P < 0.01. **(C)** Representative example of the effect of UII on prepro-UII and UT protein expression detected by Western blot of U87, U251, 8MG, and 42MG or hCMEC/D3 cells exposed to UII (10^–9^ M, 24 h) in the absence of FBS. For UT, a doublet of bands (∼42–53 kDa) and a higher molecular mass (∼70 kDa) correspond to human UT as previously shown (Lecointre et al., 2015, Supplementary Material). β-tubulin antibody was used as protein loading control.

The UII/UT system was first characterized on different glial tumors from patient tumor samples obtained after a first tumor resection or a small size biopsy (tumor bank of Haute-Normandie, France) by immunohistochemistry. These glial tumors were presented according to WHO 2008 histopathological classification, ranging from astrocytoma (AII/III), oligodendroglioma (OII/III), or oligoastrocytoma (OAII/III) to highly aggressive glioblastoma multiform (GBM, IV) based on morphological features and associated with a very poor prognosis (Xu et al., 2015). Since 2016, grade II diffuse and grade II anasplatic astrocytoma have been divided into mutated and wild-type IDH1/2 even if the great majority falls into IDHmut; astrocytomas, grade II oligodendroglioma are IDHmut and 1p/19q codeleted while GBMs are most often represented by GBM IDHwt (90% of cases) (Louis et al., 2016). Sixty patients were included retrospectively and prospectively, and tumor grades and molecular signatures were described according to the WHO 2016 classification in Table 2. The anatomic sites of the tumors were supratentorial cortex (49/60, 81%), basal ganglia (1/60, 2%), brainstem (1/60, 2%), intramedullary (1/60, 2%), cerebellar hemisphere (6/60, 10%), and vermis (2/60, 3%) (Table 2). Immunohistochemical analysis showed a higher expression of the UII/UT couple in astrocytomas (AII/III) compared with oligodendrogliomas (OII/III) when positive cells were >50% (Figure 1A). In GBMs, strong localized expression of UII and UT was observed in vascular and perinecrotic areas, in immunoreactive zones for respectively, the mesenchymal marker α-SMA-positive cells and for the carbonic anhydrase 9 (CA9) (Figure 1B), suggesting links between tumoral pericytes and hypoxia. From consecutive sections of non-tumor CNS tissue, the UII and UT immunostaining appeared in gray matter (GM), in particular in neurons instead of oligodendrocytes (peri-neuronal satellitosis) and in the white matter (WM) (Figure 1C). Score analysis of the UII and UT staining indicated a gradual increased expression with the grade, from grade I (PA) to grade IV (GBM), and also from OII/OIII, OAII/III, and AII/III (Figures 1A,D) reaching significant scores in GBM compared with PA (Figure 1D). As shown in Figure 1E, the vascular density and diameter were significantly increased in anaplastic astrocytoma and GBM while vessel defaults in circularity were shown altered in GBM. The expression levels of UII and UT were significantly correlated with increased vascular density, mainly in high-grade tumors (Figure 1E) and UII was also correlated with UT (Figure 1F) (Pearson coefficient, *P* < 0.001). Progression-free survival curves of 20 patients with primary GBM indicated that high expression of the urotensinergic components were significantly correlated with early recurrence (UII, score > 11.5/18; UT, score > 12.5/18) and were correlated with a median survival of 224 days when any of the UII and UT are highly expressed (Figure 1F). Expression mRNA level of UTS2 encoding UII, UTS2R encoding UT, and UTS2D encoding URP was also tested from TCGA (Figure 2). For each gene, profiles of normalized expression were extracted from 66 glioma samples presented in Figure 2A according to the grading classification. UTS2 was found to be significantly more expressed in astrocytoma and mostly GBM, suggesting an association with high-grade gliomas. As observed in the histopathological classification, UTS2R and UTS2D were equally expressed in IDHwt or IDHmut tumors, whereas UTS2 displayed a higher expression in IDHwt gliomas, suggesting an association with malignancy (Figure 2B). Within the four groups of GBM along with the Verhaak classification (Verhaak et al., 2010), UTS2R (UT) and UTS2D (URP) were expressed in all groups of GBM, but importantly, UTS2 (UII) exhibited a net higher expression in the mesenchymal subgroup, suggesting that UII may be associated with glioma prognosis (Figure 2C). UTS2R and UTS2 mRNA levels from TCGA showed that patients having high UTS2R or UTS2 expression exhibit significantly lower mean survival probability, while no difference was observed for UTS2D (Figure 2D). These data establish a relationship between UII, and also UT expression in GBM, mainly with the hypoxic/vascularized state of the more aggressive GBM. QPCR analyses of mRNAs encoding UII, URP, and UT in the SW1088 (anaplastic astrocytoma) and in U87, U251, 8MG, and 42MG GBM cell lines here established that expression levels were non-homogeneous and higher for UTS2R in 8MG and 42MG, UTS2 in U87, and UTS2D in SW1088 (Figure 3A). To verify a possible autocrine/paracrine mechanism in which UII may in turn induce UTS2 or UTS2R expression encoding UII and UT, we evaluate 24-h GBM cells as well as a human EC line hCMEC/D3 exposed to UII (10^–9^ M). UII stimulated the expression of UII at least in part by U87 and 8MG as confirmed by the qPCR and Western blot analysis, but not in EC constitutively expressing UII and UT (Figures 3B,C).

### UT Mediates Motility of GBMs and Endothelial Cells and Stimulates Tubulogenesis *in vitro*

**FIGURE 4:**
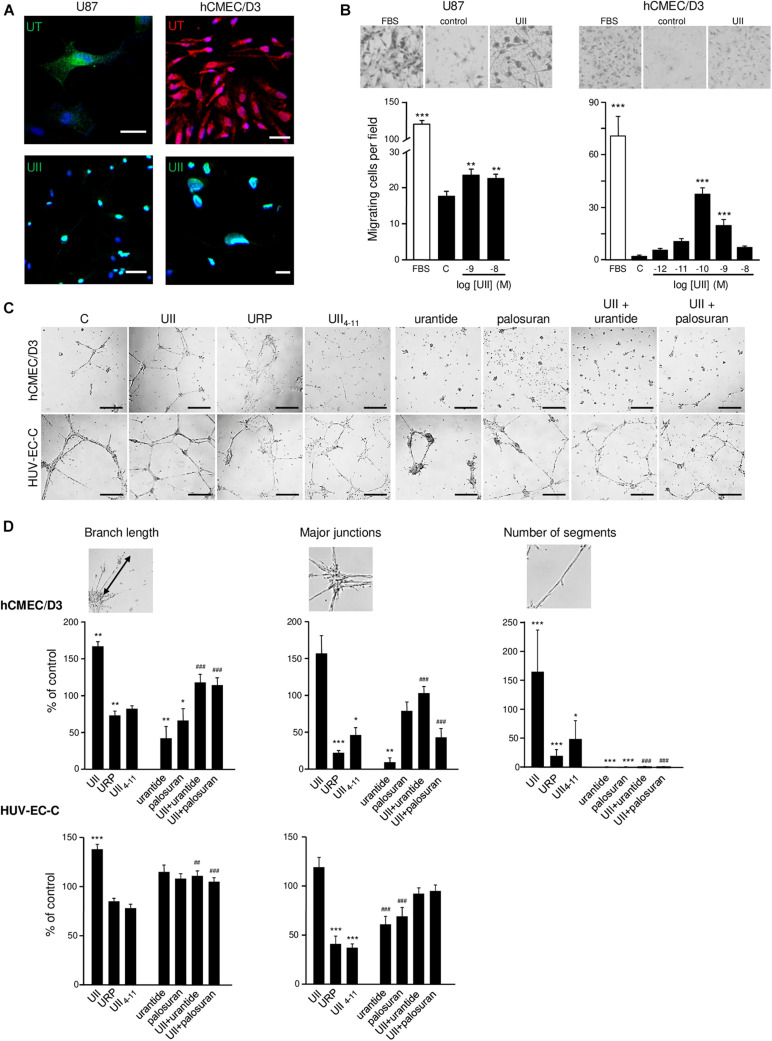
*In vitro* effect of urotensinergic ligands on human glioma and endothelial cell migration and tubulogenesis. **(A)** Microphotographs of UT and UII staining on the GBM cell line U87 and EC hCMEC/D3 from human origins. Scale bar, 20 μm. **(B)** Representative fields (*Top panel*) of migrating U87 and hCMEC/D3 cells quantified from the Boyden chamber assay and quantification of U87 (*Left*) and hCMEC/D3 (*Right*) cell number in the absence or the presence of FBS 10% or UII (10^–12^, 10^–11^, 10^–10^, 10^–9^, or 10^–8^ M). **(C)** Microphotographs of the tubulogenesis assay illustrating *in vitro* properties of UT agonists (UII, URP, and UII_4__–__11_), biased ligand (urantide), or antagonist (palosuran) on angiogenic features of hCMEC/D3 and HUV-EC-C cells. Scale bars, 400 μm. **(D)** Data are presented as the% of control ± SEM from six independent experiments, and branch length, major junctions, and segment number were quantified and compared to control conditions. Statistical significance of treatments vs. control condition was assessed with one-way ANOVA with Dunnett *post hoc* test. *ns, non-significant;* *P < 0.05; **P < 0.01; ***P < 0.001. Statistical significance of co-treatments (UII + urantide; UII + palosuran) vs. UII alone was conducted with Student *t*-test. ^##^*P* < 0.01; ^###^*P* < 0.001.

To examine the role of UII on glioma tumorigenesis, we used U87 GBM cells, endogenously expressing UII and functional UT (Figure 4A). We confirmed that UT activation by UII (10^–9^ and 10^–8^ M) leads to chemotactic migration in Boyden’s chamber assay (Figure 4B) and did not alter cell density after a 48-h treatment (Supplementary Figure 1A). We confirmed that hCMEC/D3 expressed UT at the plasma membrane and UII in the cytoplasmic and perinuclear compartments (Figure 4A). UII (10^–10^ and 10^–9^ M) stimulated hCMEC/D3 cell migration (Figure 4B) with no measurable effects on hCMEC/D3 or HU-VEC-C cell density/proliferation (Supplementary Figures 1B,C). These observations were suggestive of proinvasive and angiogenic capacities of the UII/UT system. We then tested the ability of the UT endogenous agonists UII and URP, the shorter synthetic peptide analog referred to as a full agonist UII_4__–__11_, the UT antagonist/biased ligand urantide (Camarda et al., 2004; Brulé et al., 2014), and the UT antagonist palosuran (Clozel et al., 2004; Table 1) to promote tubular organization of hCMEC/D3 and HUV-EC-C cells (Figure 4C). Only UII was able to enhance the endothelial network complexity in both hCMEC/D3 and HUV-EC-C, whereas URP and UII_4__–__11_ (10^–7^ M) disrupted EC associations (Figure 4D). The proangiogenic properties of UII were mainly relayed by EC collective motility, as demonstrated by significant increased amount of endothelial junctions and branches, resulting in a much more intensified and complex tubular structure of hCMEC/D3 and HUV-EC-C (Figures 4C,D) and increased segments and polygons of hCMEC/D3 (Figure 4D). Urantide (10^–6^ M) drastically counteracted these endothelial associations and branch length mainly in hCMEC/D3, whereas palosuran (10^–6^ M) did not significantly modify basal tubulogenesis (Figure 4D). Urantide and palosuran systematically prevented UII-induced tubulogenic effects (Figure 4D).

### Chemoattractant Function of UII Toward Proangiogenic Cells *in vivo*

**FIGURE 5:**
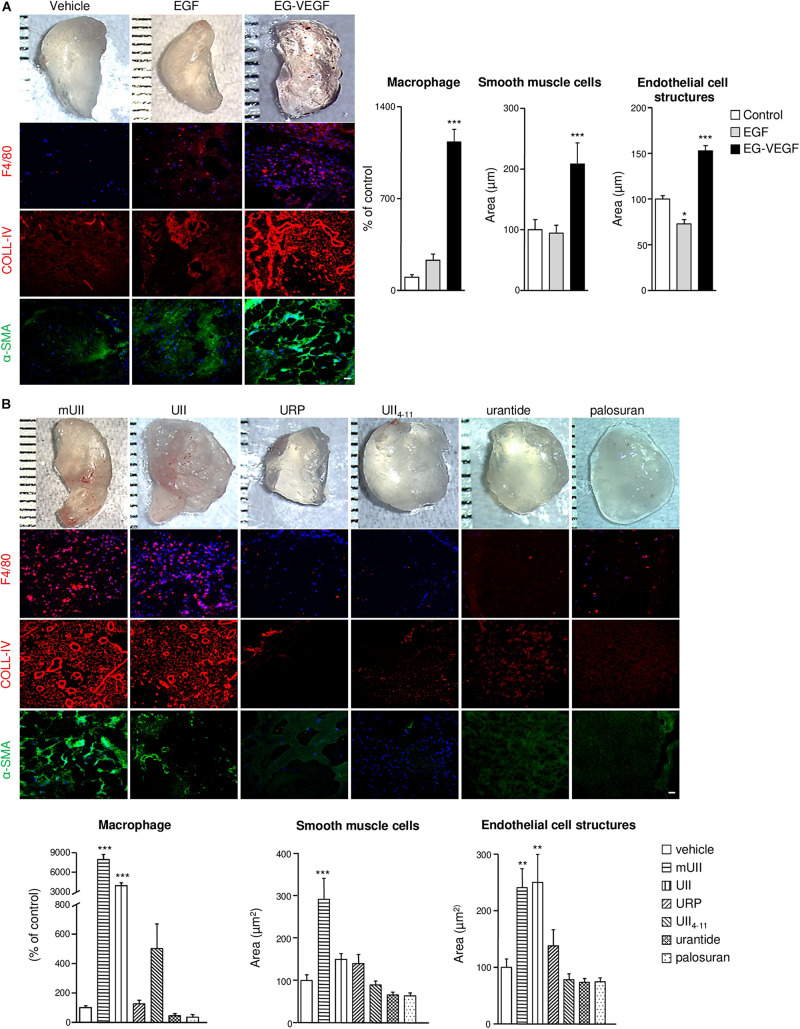
*In vivo* chemoattractant effects of the urotensinergic ligands toward pro-angiogenic cells in Matrigel plugs. **(A,B)** Macrophotographs of resected Matrigel plugs (200 μl) containing vehicle (PBS), EGF (500 ng), EG-VEGF (500 ng) **(A)** or UII, mUII, URP, UII_4__–__11_ (50 ng, each), urantide (50 ng), or palosuran (50 ng) **(B)** 3 weeks after implantation in C57B/l6 mice. Matrigel invasion was detected by immunohistochemical analysis of macrophages (F4/80, red), EC matrix collagen-IV (COLL-IV, red), and smooth muscle cells (α-SMA, green). Scale bars, 40 μm. Quantification of macrophage number or smooth muscle cell and EC structure areas. Data are presented as percentage of control ± SEM. *P < 0.05; **P < 0.01; ***P < 0.001 from at least four independent experiments. Statistical significance is given by one-way ANOVA with Dunnett *post hoc* test vs. control.

The potential proangiogenic properties of the urotensinergic system were then investigated *in vivo*, *via* Matrigel plugs subcutaneously implanted in C57B/l6 mice. To validate the assay, liquid Matrigel containing either EG-VEGF or EGF (500 ng/ml, each) was injected in mice for 3 weeks. Resected sponges revealed blood drops or fully formed blood vessels (Figure 5A) and immunopositive cells for the macrophage F4/80, endothelial basal membrane collagen-IV (COLL-IV), and α-SMA markers (Figure 5A). Matrigel sponges containing UII or the murine sequence of the peptide (mUII) (50 ng/ml, each) exhibited similar hemorrhagic drops (Figure 5B). Quantification showed a marked stimulation of macrophage invasion and of capillary-like structures, formed by the association of endothelial and smooth muscle cells (Figure 5B). URP and UII_4__–__11_ (50 ng/ml, each) or urantide and palosuran (50 ng/ml, each) failed to chemoattract cells into the plugs (Figure 5B). These results indicate that the urotensinergic system is involved in angiogenesis *in vivo* and that available drugs may constitute anti-angiogenic compounds targeting UT.

### The Urotensinergic System Is Required for GBM Growth Interfering With Mouse Survival

**FIGURE 6:**
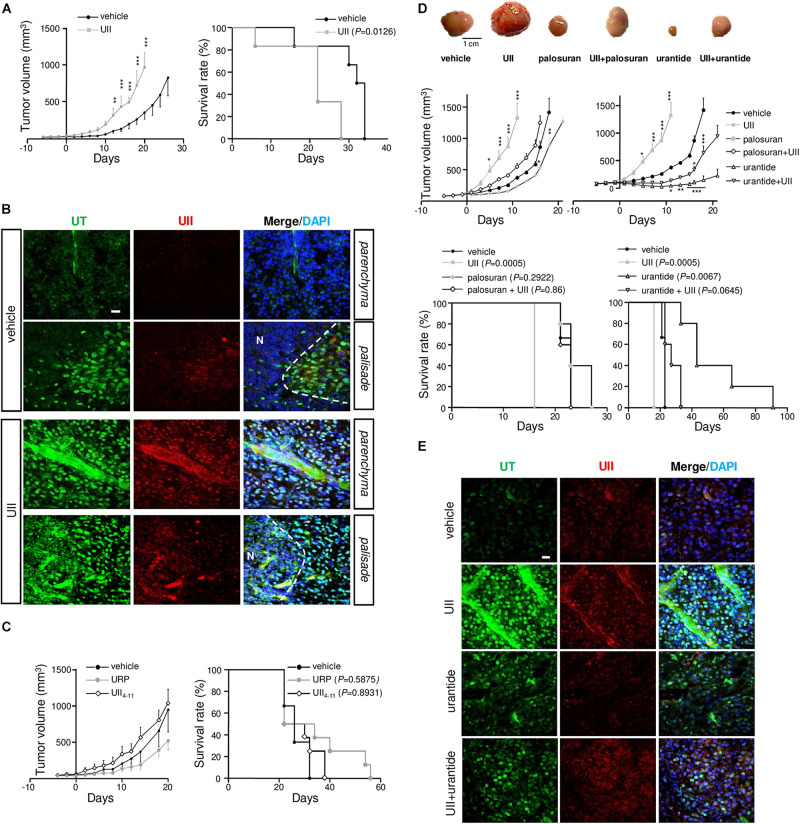
Effects of the urotensinergic system on glioma development *in vivo.* **(A)** Tumor growth (*left*) and survival (*right*) of *Swiss Nude* mice transplanted with U87 cells. When tumor reached 100 mm^3^, intratumoral injections of vehicle (saline) or UII (0.1 μg) were performed daily and tumor growth or median survival of treated mice was measured. **P < 0.01; ***P < 0.001. Median survival: vehicle (*n* = 7), 33 days; UII (*n* = 7), 22 days (*P* = 0.0126). **(B)** Microphotographs of UT (green) and UII (red) expression in vehicle (PBS)- and UII-treated U87 xenografts. UII and UT were expressed in tumor parenchyma, in perinecrotic (N) areas and in vascular structures. **(C)** Tumor growth (*Left*) and mice survival (*Right*) during treatments with vehicle, URP, or UII_4__–__11_ (0.1 μg, each) of xenografted U87. Median survival: vehicle (*n* = 4), 22 days; URP (*n* = 4), 28 days (*P* = 0.5875); UII_4__–__11_ (*n* = 4), 26 days (*P* = 0.8931). **(D)** Top panel, typical gallery of representative example of U87 xenografts treated with vehicle, UII (0.1 μg), palosuran (1 μg), UII + palosuran (0.1/1 μg), urantide (1 μg), and UII + urantide (0.1/1 μg). Middle panel, tumor growth during the different treatments. *P < 0.05; **P < 0.01; ***P < 0.001. Bottom panel, mice survival during the different treatments. Median survival: vehicle (*n* = 10), 23 days; UII (*n* = 10), 16 days (*P* = 0.0005); palosuran (*n* = 10), 23 days (*P* = 0.2922), UII + palosuran (*n* = 9), 23 days (*P* = 0.86), urantide (*n* = 10), 43 days (*P* = 0.0067), UII + urantide (*n* = 10), 27 days (*P* = 0.0645). **(E)** Immunolabeling of UT (green) and UII (red), and tumor distribution in vehicle, UII, urantide, and UII + urantide-treated U87 xenografts. Data presented as mean ± SEM. Animal survival was analyzed with Kaplan–Meier method, using the log-rank test for comparison. Statistical significance for tumor growth in **(A,C,D)** was given by using two-way ANOVA with Bonferroni *post hoc* test comparison with vehicle. Cell *nuclei* stained with DAPI (blue). Scale bars: 20 μm.

To investigate the involvement of the urotensinergic system in angiogenesis and tumorigenic process in GBM, daily intratumoral injections of UII on U87 xenograft tumors were performed when tumors reached ≈100 mm^3^. Exogenous administration of UII (2.9 ng/kg) significantly accelerated tumor growth (Figures 6A,D) without affecting the body weight (Supplementary Figure 2). The median survival of animals receiving intratumoral injections of UII decreased compared with vehicle (Figures 6A,D). Vehicle or UII-tumor xenografts expressed UII and mostly UT in perinecrotic areas (pseudopalisadic area) and in vascular components (Figure 6B). URP (2.9 ng/kg) and UII_4__–__11_ (2.9 ng/kg) did not significantly modify GBM tumorigenic growth as well as mouse median survival (Figure 6C).

Animals receiving intratumoral administration of palosuran featured diminished tumor growth but no significant extended survival (Figure 6D, *P* = 0.2922). Co-administered with UII, palosuran partially prevented the UII-evoked stimulation of tumor growth (Figure 6D). The biased UT ligand urantide showed a strong inhibitory effect on tumor growth by maintaining significantly smaller tumor volumes for an extended period (Figure 6D), whereas the animal survival was markedly increased. When added at a 10-fold higher dose (Supplementary Figure 3A), or when tumors reached a large volume (>500 mm^3^, Supplementary Figure 3B), urantide exhibited a significant anti-tumoral effect. Also, UII-treated xenografts expressed UII and UT in GBM and in vascular components (Figure 6E), while urantide prevented UII-promoting UII and UT expression (Figure 6E).

### The Urotensinergic System Is Involved in GBM Cell Proliferation, Necrosis, and MMP-2/9 Activation *in vivo*

**FIGURE 7:**
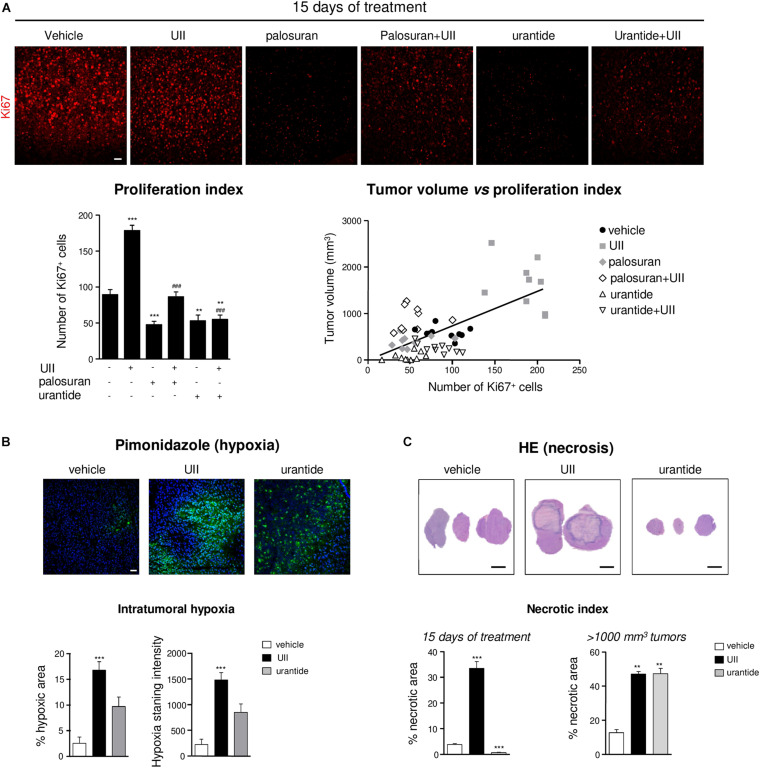
Effects of the urotensinergic system on cell proliferation, hypoxia, and necrosis in U87 GBM. **(A)** Representative microscopic fields (*Top panel*) of U87 xenografts immunostained with an antibody directed against the proliferation marker Ki67 (red). Bottom left, quantification of the number of proliferative cells (Ki67^+^) after 15 days of daily intratumoral injections of the UT ligands; bottom right, linear regression of tumor volume vs. proliferation index. Statistical significance of vehicle versus UII, palosuran, urantide, or co-treatments at day 15. **P < 0.01; ***P < 0.001. Statistical significance of UII versus co-treatments at day 15. *###P* < 0.001. A significant positive correlation was given by the Pearson coefficient correlation *r* = 0.6579 (*P* < 0.0001). Scale bars: 40 μm. **(B)** Top, microscopic fields of U87 xenografts of tumoral hypoxia revealed by pimonidazole labeling (green) after 15 days of daily intratumoral injections of vehicle, UII (0.1 μg), or urantide (1 μg). Bottom, quantification of pimonidazole stained area (*Left*) and intensity (*Right*). Scale bars: 40 μm. *n* = 4 in each treatment groups. ***P < 0.001. **(C)** Top, H&E necrotic area coloration after 15 days of daily intratumoral injections of vehicle, UII (0.1 μg), or urantide (1 μg). Bottom, quantification of the necrotic area after 15 days (*left*) or after tumors reached 1000 mm^3^ (*Right*). Scale bars: 2 mm. *n* = 6 in each treatment groups. **P < 0.01; ***P < 0.001.

We next tested whether U87 xenograft treatments result in UT-regulating GBM proliferation. After a 15-day treatment, UII significantly increased the proliferation index, measured by the immunostaining of the proliferation marker Ki67 (Figure 7A). Palosuran and urantide significantly diminished endogenous cell proliferation by themselves, and UII-induced proliferation was also prevented by palosuran or urantide (Figure 7A, *bottom left*). Accordingly, a significant correlation (Pearson’s *r* = 0.6579, *R*^2^ = 0.4328, *P* < 0.0001) between the proliferation index and tumor volume was found at 15 days (Figure 7A, bottom right).

Rapid proliferation within glioma outstrips their blood supply likely leading to intratumoral necrosis and induction of hypoxia likely involving HIF-1α and HIF-2α overexpression (Supplementary Figure 4). We then investigated the distribution of the hypoxic marker pimonidazole in U87 xenografts after a 15-day treatment or when tumors reached 1,000 mm^3^. In UII-treated tumors, the size of hypoxic areas (green) was strongly increased, indicating the hypoxic status of UII-treated tumors (Figure 7B). After urantide treatment, a similar tendency can be observed without significance. In GBM, the sustained hypoxia is classically followed by necrosis. The size of necrotic area was quantified after H&E coloration on 15-day-treated and on large tumors (>1,000 mm^3^). UII strongly increased the size of necrotic areas (Figure 7C), whereas urantide evoked a significant decrease of necrosis at day 15, and finally showed a necrotic index resembling those of the UII-treated tumors after tumor relapse (Figure 7C).

**FIGURE 8:**
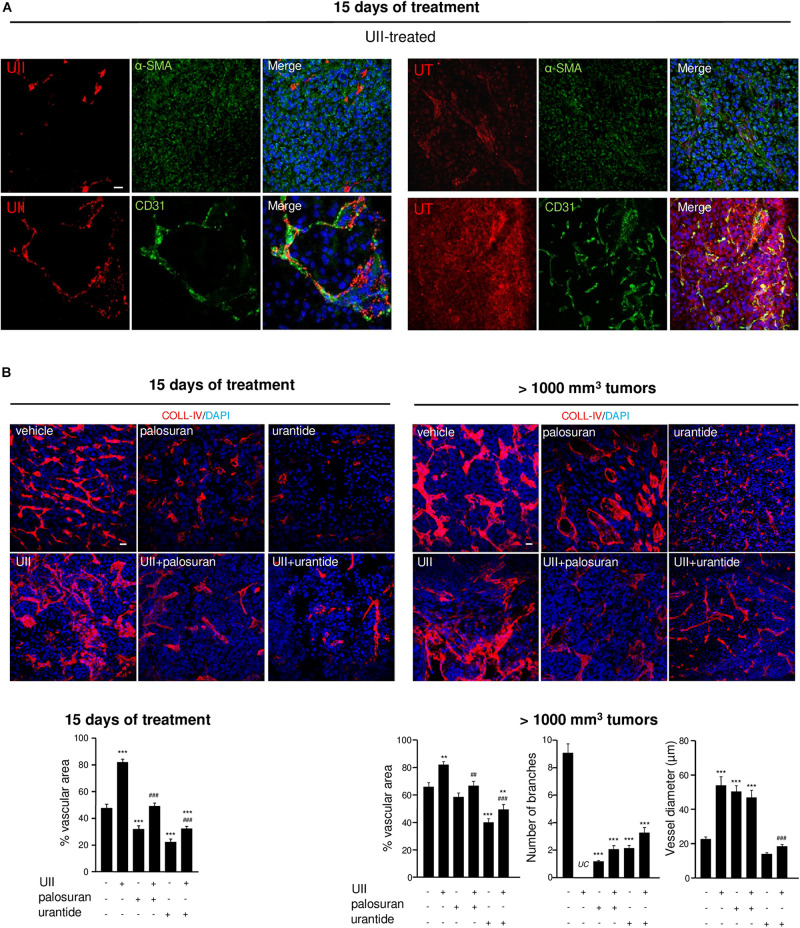
Effects of the urotensinergic system on angiogenesis in U87 GBM. **(A)** UII (Top) and UT (Bottom) (red, each) immunostainings co-localized with VSMC and EC detected with anti-αSMA and anti-CD31 (green), respectively, in U87 xenografts. **(B)** COLL-IV (red) labeling in U87 tumors after 15 days of different treatments (*left panel*) or after tumors reach 1000 mm^3^ (*right panel*). Quantification of intratumoral angiogenesis (vascular area) after 15 days of treatments or after tumors reach 1000 mm^3^ (vascular area, number of branches and vessel diameter) as percentage of control ± SEM. **P < 0.01; ***P < 0.001 vehicle vs. treatments; ^##^*P* < 0.05; ^###^*P* < 0.001; UII versus UII + palosuran or UII + urantide, from at least four different tumors in each group. Statistical significance was given by one-way ANOVA followed by Dunnett *post hoc* test. *UC*, uncountable.

In UII-treated tumors, capillaries appeared co-stained with anti-UII and anti-CD31, suggesting that endothelial compartment also serve as a reservoir of UII production (Figure 8A, left panel). UT was highly expressed in both glioma and EC, but not in α-SMA-positive structures (Figure 8A, right panel). The angiogenic status of xenografts under UII, urantide, or palosuran treatment after 15 days of daily injections and when tumors have reached 1,000 to 1,500 mm^3^ was investigated. At 15 days, exogenous UII strongly altered the blood vessel architecture, with tortuous and disorganized vascular networks (Figure 8B, left). Palosuran or urantide markedly diminished the blood vessel density (Figure 8B, left). In >1,000 mm^3^ tumors, UII maintained a strong angiogenic status whereas palosuran but mostly urantide decreased the basal vascular density and number of branches (Figure 8B, right), only urantide being able to reduce capillary-like structure diameter (Figure 8B, right). These suggest that urantide acts as a potent antagonist of the murine UT expressed on vascular components of host mice, to evoke a strong angiogenic activity.

**FIGURE 9:**
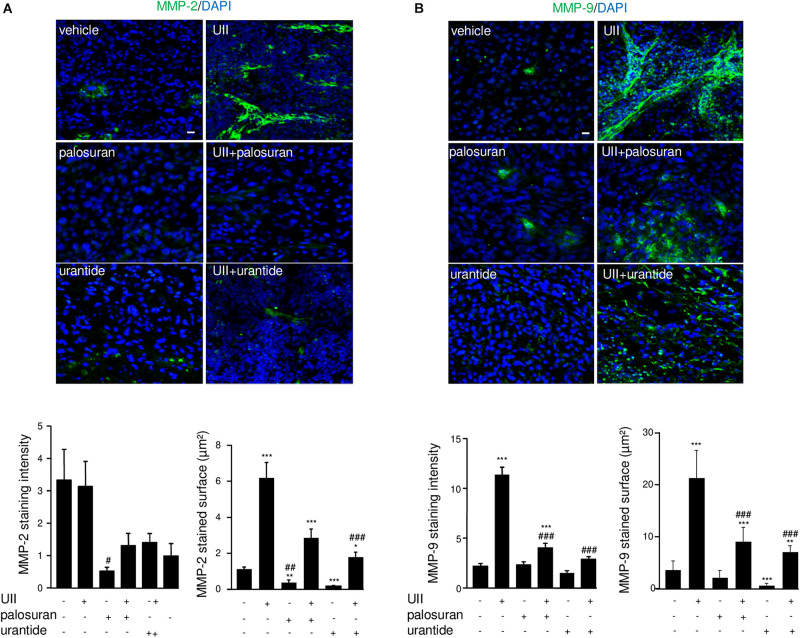
Effects of the urotensinergic system on metalloprotease activation in U87 GBM. **(A,B)** MMP-2 (green) and quantification of labeled intensity and surface **(A)** or MMP-9 staining (green) and quantification **(B)** of labeled intensity and surface. Statistical significance was given by one-way ANOVA followed by Dunnett *post hoc* test. *P < 0.05; **P < 0.01; ***P < 0.001 vehicle *versus* treatments. ^#^*P* < 0.05; ^##^*P* < 0.01; ^###^*P* < 0.001 UII vs treatments. Cell *nuclei* stained with DAPI (blue). Scale bars: 20 μm.

Glioblastoma growth, hypoxia, and angiogenesis have been shown to be associated with mesenchymal marker expression including MMPs, and more especially MMP-2 during blood vessel reorganization and MMP-9 during invasion (Xu et al., 2015). As illustrated in Figure 9A, MMP-2 was mainly localized in large capillary-like structures and showed significant higher surface covered after treatment with UII. The proinvasive MMP-9 is also observed in large blood vessels and surrounding tumor cells, with intense immunofluorescence in UII-treated xenografts (Figure 9B). A similar trend was found *in vitro*, in hCMEC/D3 with higher MMP9 gene expression upon UII treatment (Supplementary Figure 5). Palosuran and, more efficiently, urantide markedly prevented MMP-2 and MMP-9 expression whereas urantide significantly counteracted the UII-evoked MMP-2 and MMP-9 activations (Figures 9A,B). These observations suggest endogenous UII-induced hypoxic and proliferative mechanisms in GBM, likely stimulating mesenchymal phenotype associated with angiogenic features.

### UII Stimulates αvβ Integrin Expression and ^99m^Tc-RGD Binding in U87 GBM Xenografts

**FIGURE 10:**
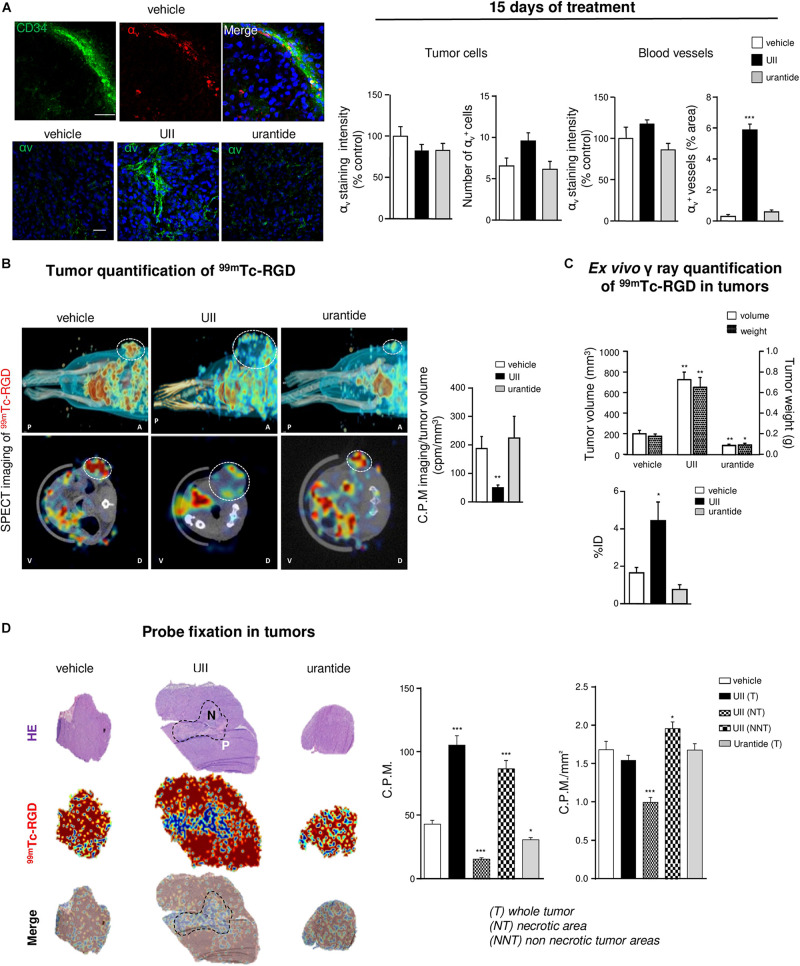
*In vivo* imaging of the urotensinergic system-associated integrin expression and angiogenesis. **(A)** *Left*, U87 xenografts neo-vessels stained with antibodies directed against the endothelial precursor marker CD34 (green) and α_V_ integrins (red) after 15 days when daily treated with vehicle (*top panel*) or α_V_ integrins (green) in vehicle-, UII (0.1 μg)-, or urantide (1 μg)-treated xenograft after 15 days treatment. *Right*, Quantification of α_V_ staining in tumor xenografts after 15 days of different treatments in tumor parenchyma and vascular compartment. *P < 0.05; **P < 0.01; ***P < 0.001; vehicle vs. treatments. Cell *nuclei* stained with DAPI (blue). Scale bars: 20 μm. **(B)** Representative MicroSPECT imaging of the ^99m^Tc-RGD integrin ligand (red shades) binding in living tumors (circled by white dots), in antero-posterior (*A-P, top*), or dorso-ventral (V-D, *bottom*) positions and quantification of tracer incorporation in tumors (*right*) after 15 days of treatment. **(C)** Quantification of tumoral volume/weight and ^99m^Tc-RGD γ ray emission in resected tumors, after 15 days of treatment (left, *n* = 6 in each group of treatment). ID, injected dose. **(D)** *Left*, representative fields of necrotic staining with H&E (*top*), ^99m^Tc-RGD tracer binding (*middle*), and merged pictures (*bottom*) from consecutive slices of tumors acquired by β imaging. Scale bar: 2 mm. Right, Quantification of ^99m^Tc-RGD in entire histological tumor sections (tumor, T) or when discriminating necrotic tumor areas (NT) in UII-treated tumors (non-necrotic tumor, NNT). *n* = 6 in each treatment groups. *P < 0.05; ***P < 0.001. Statistical significance in **(A–D)** experiments was given by one-way ANOVA with Dunnett *post hoc* test comparison with vehicle.

Angiogenesis and mesenchymal phenotype are accompanied by integrin reactivation. At 15 days, it was observed that αv-immunolabeled structures expressed the EC progenitor CD34 marker, more specifically in UII-treated tumors (Figures 10A,B, left). *In vitro*, UII favored the expression of the ITGAV gene mRNA encoding alphav integrin from at least in part U87 and 8MG GBM cells (Supplementary Figure 5). The αvβ3 integrins were previously investigated as targets for direct molecular imaging of tumor angiogenesis with SPECT as they are considered as a key marker of activated EC (Schnell et al., 2009). Here, we used a ^99^Technetium tracer containing the RGD (arginine, glycine, aspartate, ^99^Tc-RGD) binding motif already validated (Rylova et al., 2014) to image αvβ3 expression in GBM *in vivo* (Supplementary Material and Supplementary Figure 6). In U87 xenografts, UII stimulated tumor growth (Figures 10B,C) but paradoxically provoked a decrease of RGD tumor uptake after 15 days of treatment (Figure 10C). The *ex vivo* γ-ray quantification of the total tumor showed ^99^Tc-RGD capture enhanced in UII- and reduced in urantide-treated tumors, respectively, mainly due to the tumor size. From merged H&E staining and the ^99^Tc-RGD distribution, it was observable that 10% of the control tumors and whole UII-treated tumors were necrotic, respectively. Analysis of the ^99^Tc-RGD capture in the tumor (T), the necrotic tumor (NT), or the non-necrotic tumor (NNT) revealed that UII-treated tumors exhibited more RGD binding around the necrotic area (Figure 10D), suggesting increase of integrin expression and potentially tumor angiogenesis.

## Discussion

Angiogenesis has been shown to play a key role in the multi-step formation of GBM and results from a complex multicellular communication between glioma, endothelial, inflammatory, and/or reactive glial cells (Hanahan and Coussens, 2012). However, anti-angiogenics including bevacizumab can prolong the progression-free survival in patient with recurrent GBM (Lai et al., 2011) but led to a dramatic progression likely *via* exacerbated hypoxic and mesenchymal acquired status (Bergers and Hanahan, 2008; Keunen et al., 2011). Therefore, targeting vasoactive peptide receptor behaving as angiogenic chemokines involved in angiogenesis, inflammatory cell attraction, and invasion of GBM cell lines should constitute an original and promising target system. Here, we report that the peptide UII and its receptor UT, expressed in malignant high-grade gliomas, promote angiogenesis and tumor vascular abnormal phenotype *via* up-regulation of mesenchymal factors including αvβ integrins and MMP-9, accompanying tumor growth, proliferation, and hypoxia/necrosis. This UII/UT system expressed in the U87-xenografted GBM model is shown to be efficiently targeted by biased/antagonist UT ligands leading to tumor growth inhibition by, at least in part, reducing angiogenesis.

From TCGA, we show that UTS2 encoding UII is more expressed in high-grade astrocytic gliomas including GBMs and, within GBM, appears to be upregulated in the mesenchymal subclass of the Verhaak classification. A significant positive correlation between UT and UII expression in glioma samples also suggest paracrine/autocrine mechanisms within the GBM tumor bulk. Interestingly, glioma patients expressing the highest levels of UT and mainly UII mRNA had significantly shorter survival durations than cases with lower expressions; this prognostic cue can be associated with the strong expression of UII and UT in the hypoxic/vascular area of GBM samples, the positive correlation found between UII and UT expression, and the density of the abnormal vascularization. Indeed, inflammatory processes and/or hypoxic microenvironment within the tumor or neighboring normal tissues likely result in secretion by GBM cells of immunomodulatory cytokines and other factors such as CSF1 (colony-stimulating factor 1), C-C/CXC motif chemokines, or GDNF (glial cell-derived neurotrophic factor), which polarize TAMs toward an immunosuppressive M2 phenotype (Chang et al., 2016; Chen et al., 2017; Wang et al., 2019). TAM may in turn induce growth factor release triggering specific transcription factors important for mesenchymal transition associated with angiogenesis (Bogoch et al., 2017). Indeed, mesenchymal GBMs are characterized by expression of chemokine ligand/GPCRs such CXCL12/CXCR4, thrombin/PAR-1, or IL-8/CXCR2-associated signaling stimulating angiogenesis (Rollins, 2006; Albini et al., 2018) or factors supporting tissue remodeling and angiogenesis such as MMP-2 or MMP-9 (Gabrusiewicz et al., 2011). While GBM expressed UII and UT more specifically in mesenchymal tumor areas, the previous description of UT expression in a majority of monocytes/macrophages and the UII-induced TAM infiltration promoting an inflammatory environment in lung cancer (Segain et al., 2007; Zhou et al., 2012) strongly support a key role of the UII/UT system in hypoxia/inflammation-induced mesenchymal transition, TAM infiltration, angiogenesis, and, as a consequence, resistance to treatment.

The pro-inflammatory cytokine function of the UII/UT system at the vascular compartment has been suggested by the UII-induced synthesis of pro-thrombotic and inflammatory markers such as PAI-1, in cultured smooth muscle cells or EC (Djordjevic et al., 2005; Cirillo et al., 2008). Here, we confirmed the expression of UT and UII by glioma cells and showed that UII induces chemotactic migration and tubulogenesis of human hCMEC/D3 and HUV-EC-C, without main impact on cell proliferation. The observed chemoattractant effects of UII on brain and umbilical ECs are in a good agreement with the first study of Spinazzi et al. (2006) establishing *in vivo* tubulogenic properties and sustains a UII-induced initiation of vessel sprouting during co-optation. These angiogenic properties were validated *in vivo* by means of the matrigel plugs, in which angiogenic compounds such as EG-VEGF allowed penetration by host cells and formation of new blood vessels. Matrigels containing urotensinergic ligands chemoattract and capture host cells, e.g., macrophages, vascular smooth muscle cells, or EC (Matsusaka and Wakabayashi, 2006; Segain et al., 2007; Park et al., 2013) signs of pro-inflammatory and pro-angiogenic properties of UII toward murine cells. Interestingly, the presupposed UT agonists URP or the short sequence peptide UII_4__–__11_ failed to promote recruitment of vascular cells *in vitro* (Table 1). Also, we describe the angiogenic potential of UT on EC *in vivo* when activated by UII but not URP or hUII_4__–__11_. Originally observed here, URP and shorter UII-derived sequences exhibit antagonistic/inhibitory properties on tubulogenesis *in vitro*, suggesting a potential endogenous biased activity of URP on UT, antagonizing UII-induced migratory function. Some distinct activities on vascular and glial functions (Prosser et al., 2008; Hirose et al., 2009; Jarry et al., 2010) would at least in part reside in the UII-mediating long-lasting effects through insurmountable binding on UT, whereas URP usually induces transient responses (Hirose et al., 2009; Desrues et al., 2012). Both URP and hUII_4__–__11_ were used as lead sequence for the design of UT synthetic drugs including antagonists. The first “peptide antagonists” of rodent UT were obtained by focusing on Lys8 of UII to develop urantide (Table 1; Patacchini et al., 2003) exhibiting on CHO or HEK cells expressing recombinant human UT residual agonist (Camarda et al., 2004) or biased activity (Brulé et al., 2014). Here, when urantide was directly tested on EC-forming tubulogenesis, a marked constitutive inhibition is observed whereas the antagonist palosuran (Table 1) remained inactive on tube formation, both preventing UII-induced angiogenesis. The urantide-inhibitory function raises the possibility of a biased function negatively regulating the UT-signaling cascades, e.g., partially Gq and internalization as previously shown (Brulé et al., 2014), specifically involved in tubulogenesis. Diebold et al. (2012) demonstrated a NOX2-containing NADPH oxidase as a source of ROS responsible for UII-induced angiogenesis (Patacchini et al., 2003), supporting the idea that UII recruits NOX2 through a mechanism involving at least in part Gq and/or internalization of UT. To dissect the involvement of the urotensinergic system in GBM growth and angiogenesis, U87 heterotopic xenografts in *Nude* mice were injected with UT “agonists” (UII or URP or hUII_4__–__11_), biased ligand (urantide) or antagonist (palosuran) alone, or in combination with UII. UII dramatically accelerates GBM growth, correlated with a significant reduced animal survival, while URP or hUII_4__–__11_ remained inactive at the same tested doses and/or exhibit tumor repressor function at a higher dose, reinforcing the notion that URP may be a naturally produced biased/competitive ligand of UT. In the same line, the short peptide urantide drastically inhibited GBM growth and promoted long-lasting survival. By comparison, the most well-studied primate UT antagonist palosuran only delayed gliomagenesis and animal death. It can be explained by the low antagonist behavior of palosuran toward rodent UT present in host cells, compared with the antagonist activity of urantide toward murine UT (Patacchini et al., 2003) combined with its biased activity on human UT (Brulé et al., 2014). As recently validated with the design of a radiolabeled DOTA-urantide binding UT injectable *in vivo* (Poret et al., 2020), UT expressed in GBM cells as well as in vascular/myeloid compartments would constitute a key pharmacological target for the design of therapeutic molecules based on the structure of urantide.

Detection of the key markers of the high-grade GBM as well as measurement of the proliferation index and hypoxic–necrotic areas provide cues of the impact of the urotensinergic system on glioma malignancy. We show that UII enhanced cell proliferation within GBM tumors, whereas palosuran and urantide inhibited this mitogenic activity, even in the presence of exogenous UII. This UII-promoting mitogenic mechanism should not result from a direct activation of glioma cells (Lecointre et al., 2015). However, anti-angiogenic treatment leads to tumoral hypoxic features and mesenchymal phenotype associated with increased proliferative capacity (Xu et al., 2015). Here, UII similarly exacerbates hypoxia surrounding large central necrotic areas, likely mimicking the adverse events of anti-angiogenics. Despite a report suggesting that UT relays activation of HIF-1α through NOX2 in endothelial cells controlling tubulogenesis *in vitro* (Diebold et al., 2012), we did not observe UII-evoked induction of HIF-1/2 in normo- or hypoxic conditions in GBM or EC *in vitro*. UII may rather configure the tumor microenvironment composed of endothelial, perivascular, and/or inflammatory cells, promoting tumor progression by mediating hypoxia, abnormal angiogenesis, desmoplasia, and/or mesenchymal characteristics. The mesenchymal components including MMP-2 and MMP-9 can contribute to tumor invasion through breaking down of basement membrane including Coll-IV (Fan et al., 2012). In agreement, the production of MMP-9 after UII treatment of lung adenocarcinoma in mice (Zhou et al., 2012) was previously described. We show that UII treatment of GBM xenografts evoked MMP-2 and MMP-9 overexpression mainly in the vascular and glioma cell compartments, respectively, and that UII stimulates MMP9 gene expression in hCMEC/D3 *in vitro*. Again, palosuran and urantide diminished and prevented UII-evoked MMPs expression, indicating that the urotensinergic system activates the vascular network during tumor progression.

Extracellular Coll-IV and membrane α_V_ integrins are known to be scaffolding adhesion molecules for angiogenesis and tumoral infiltration correlated with progression of numerous cancers (Mammoto et al., 2013). We describe and quantify α_V_ integrin patterns in glioma cells or vessels and establish a marked increased expression in endothelium and glioma cells during the course of GBM malignancy when treated with UII. These αvβ3 integrins were previously investigated as targets for molecular SPECT imaging of tumor angiogenesis *in vivo* while they are also expressed by glioma cells (Schnell et al., 2009). We observe increased GBM uptake of ^99^Tc-RGD in the periphery of the tumor surrounding the necrotic area after UII treatment. However, *in vivo* radio-counting showed decreased incorporation within the tumor. As recently previously proposed for bevacizumab-treated GBM in mice (Rylova et al., 2014), delayed necrosis explains the decrease of RGD uptake *in vivo*. We believe UII sets up tortuous angiogenesis with enhanced integrin expression, associated with chaotic blood flow and/or interstitial pressure reducing perfusion, thereby confining RGD probe capture to the tumor peripheral ring.

**FIGURE 11:**
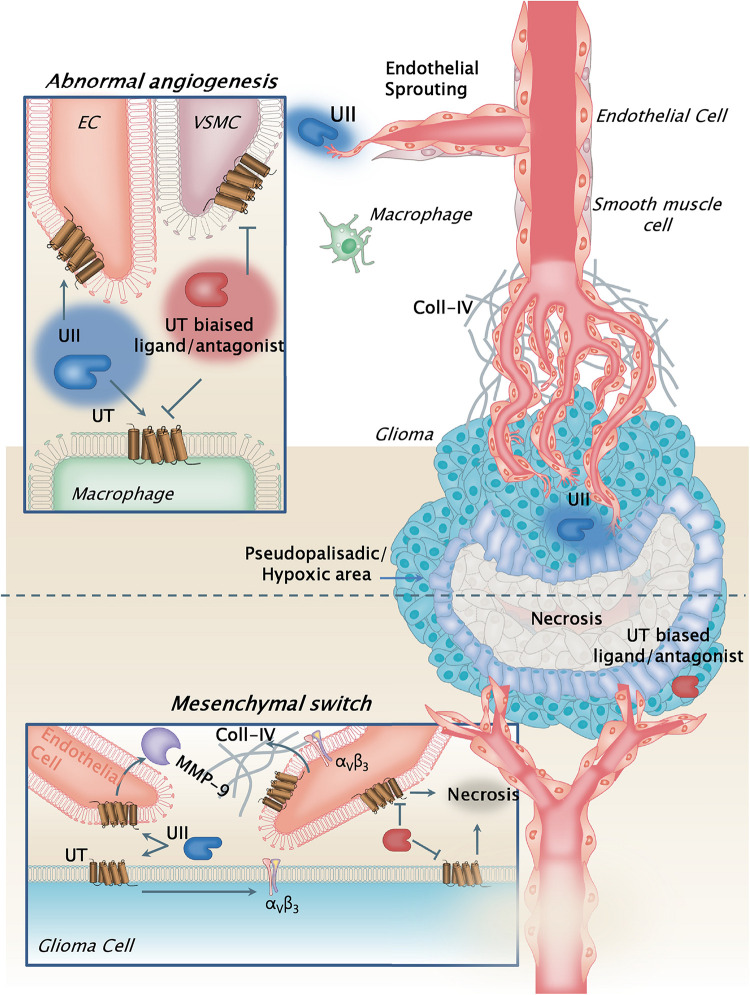
Schematic model illustrating the pleiotrope functions of the urotensinergic system during GBM malignancy. The UT receptor when expressed at both the tumoral and vascular compartments, and activated by UII through endogenous release by tumor cells, relayed accelerated tumor growth and proliferation, hypoxia, and necrosis, leading to exacerbation of the abnormal and tortuous vascularization. These processes are accompanied by metalloprotease (MMPs) release such as MMP-9 by the endothelial compartment likely degrading extracellular matrix, and by increased expression of αv(β3) integrins at least in part by GBM cells. The hypothesis of a contributing mechanism of a UII-induced macrophage tumor invasion can also be proposed, as potential cell partners in necrosis and angiogenesis. UT receptor antagonist palosuran or the biased ligand urantide would constitute a new original strategy to prevent glioma malignancy. Here, the biased ligand urantide exhibits a better multicellular anti-UT activity than palosuran both repressing angiogenesis and tumor growth, suggesting a new avenue for GBM treatment targeting the urotensinergic system.

Altogether, our study emphasizes that UII and its receptor UT are more expressed in GBMs than in low-grade gliomas, specifically in vascular and hypoxia mesenchymal areas, and play a major role in gliomagenesis *via*, at least in part, angiogenesis, promoting MMP-2/-9 and α_V_ integrin expression within the tumor bulk. Because UT may also relay glioma cell migration, this work shows the interesting opportunity provided by new class of vasoactive and chemokine peptide receptor targets controlling multicellular functions within the GBM microenvironment and involved in the mesenchymal transformation (Figure 11).

## Data Availability Statement

The original contributions presented in the study are included in the article/Supplementary Material, further inquiries can be directed to the corresponding author/s.

## Ethics Statement

The animal study was reviewed and approved by the “Comité d’Ethique NOrmandie en Matière d’EXpérimentation Animale” CENOMEXA under the National Committee on Animal Experimentation, and received the following number N/13-11-12/36/11-17.

## Author Contributions

VLJ performed main experiments, quantifications and analyses on glioma cells and endothelial cells *in vitro* (cell migration, tubulogenesis) and *in vivo* (matrigel plugs, tumor xenografts and ligand injections, animal immunohistochemistry, MicroSpect imaging). P-OG and K-PD carried out immunohistochemistry, analysis, scoring from patient clinical information, and quantitative PCR on glioma biopsies and glioma cell lines. AM analyzed *in silico* mRNA expression levels from the TCGA database and vascular characteristics from glioma patient biopsies. NP, DC, and LD maintained culture of glioma and endothelial cell lines, and performed cell proliferation studies, and Western blot for hypoxic marker expression. P-OC established and provided the hCMEC/D3 human microendothelial cell line. JH contributed to the studies on the GBM cell xenografts and intratumoral administration of urantide. F-XF, OL, AL, and FMa provided GBM biopsies and all clinical information from the Haute-Normandie tumorbank, France. RM, PB, and PV established the angiogenic RGD tracer and allowed the *in vivo* microSPECT imaging and analysis. FMo contributed to the analysis of the data and provided critical comments. PG and HC supervised the study, provided all critical analyses, and wrote the manuscript. All authors contributed to the article and approved the submitted version.

## Conflict of Interest

The authors declare that the research was conducted in the absence of any commercial or financial relationships that could be construed as a potential conflict of interest.

## Footnotes

1 http://www.bioinformatics.nl/cgi-bin/primer3plus/primer3plus.cgi/

2 https://www.cancer.gov/about-nci/organization/ccg/research/structural-genomics/tcga
