# Supplementary material for: Targeting the Urotensin II/UT G Protein-Coupled Receptor to Counteract Angiogenesis and Mesenchymal Hypoxia/Necrosis in Glioblastoma
Source: Front Cell Dev Biol. 2021 Apr 14;9:652544. doi: 10.3389/fcell.2021.652544 (PMC8079989; doi:10.3389/fcell.2021.652544)
Supplement: Supplementary file 1 [file Data_Sheet_1.PDF]

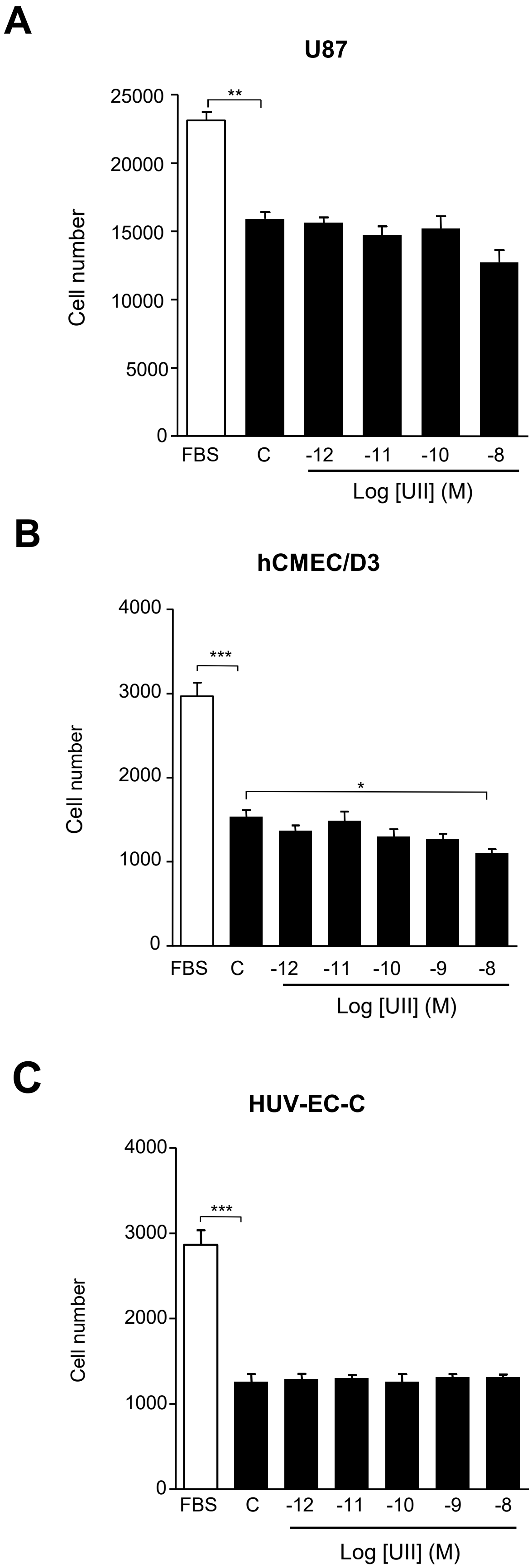

Figure S1 Le Joncour et al.

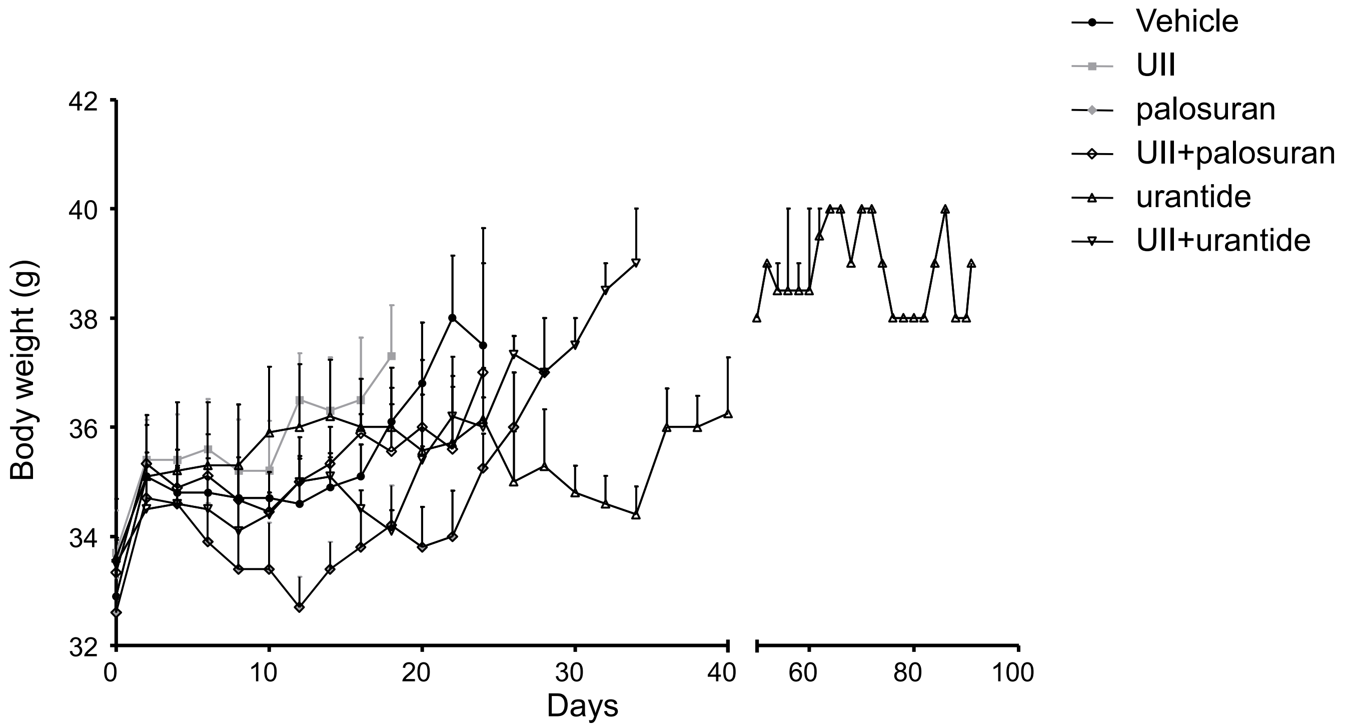

Figure S2 Le Joncour et al.

A

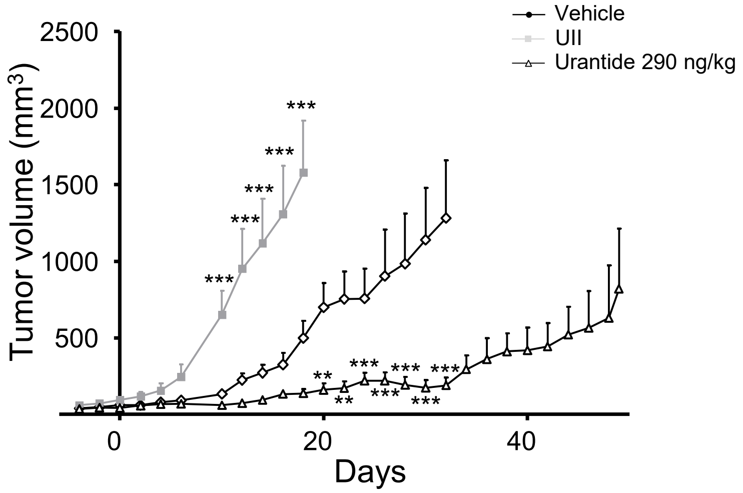
Vehicle
UII
Urantide 290 ng/kg
Tumor volume (mm3)
2500
2000
1500
1000
500
0
20
40
Days
***
**

B

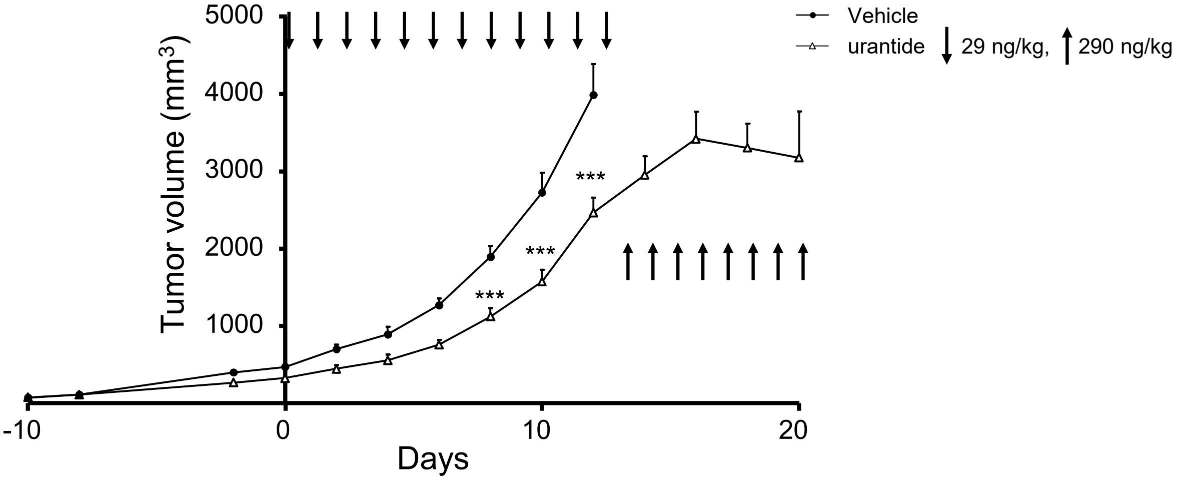
Vehicle
urantide
29 ng/kg,
290 ng/kg
Tumor volume (mm3)
5000
4000
3000
2000
1000
-10
0
10
20
Days
***

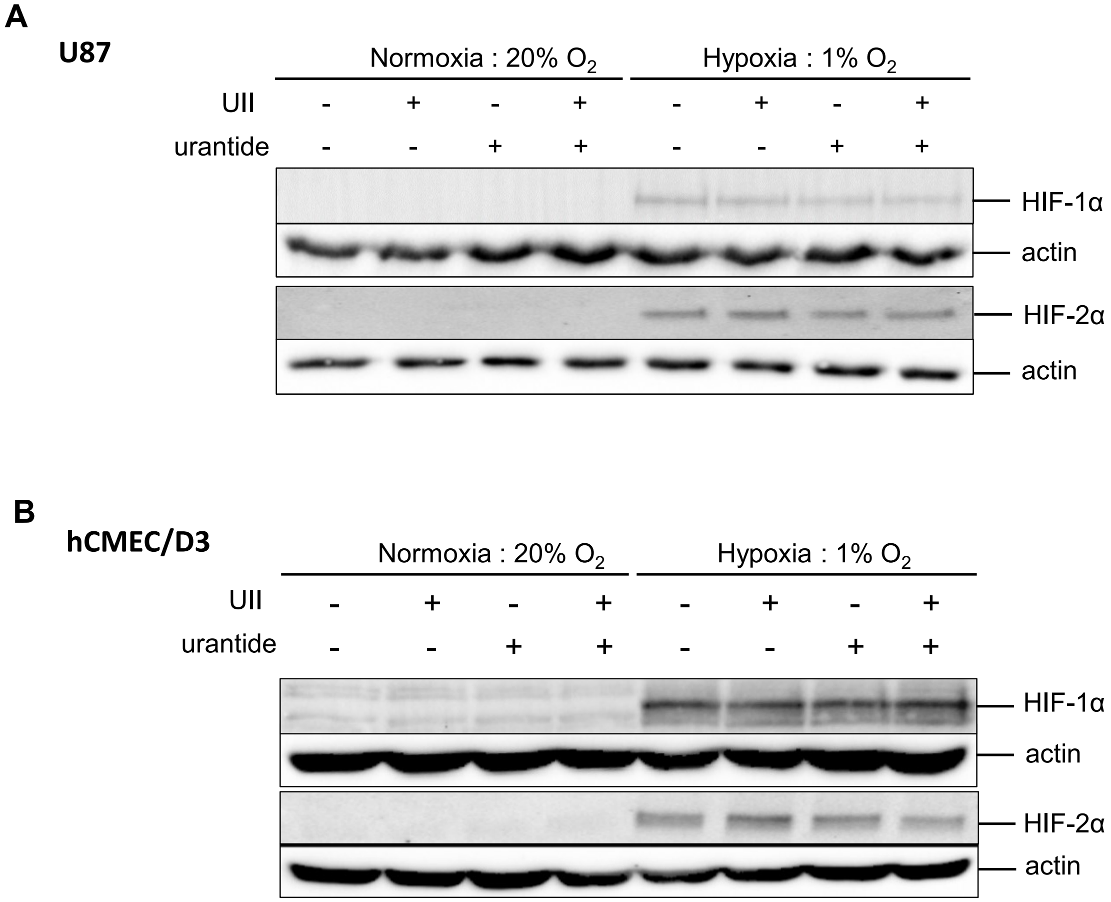

Figure S4 Le Joncour et al.

A

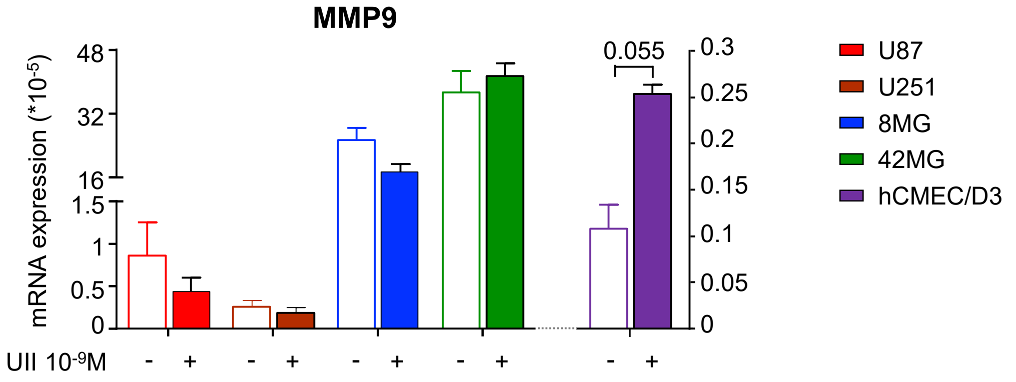

B

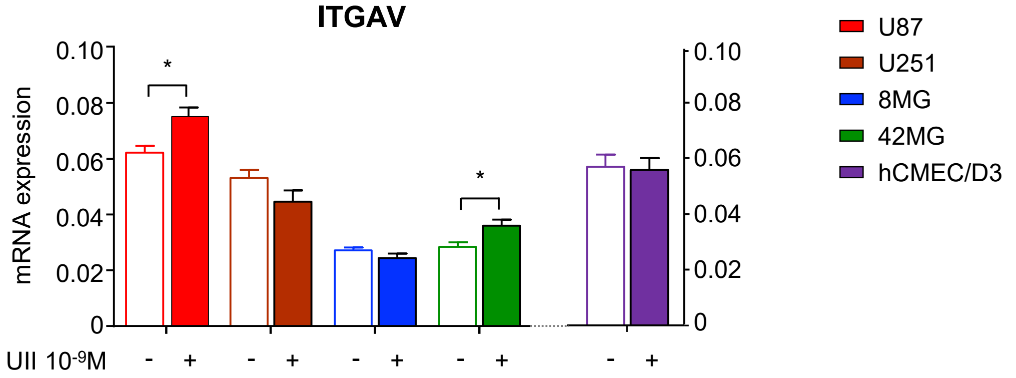

Figure S5 Le Joncour et al.

A

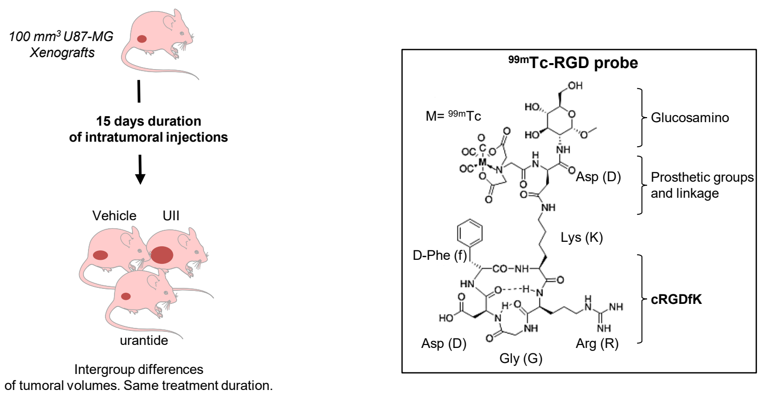

B

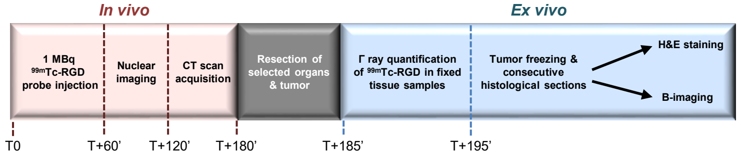

C

15 days of treatment

Treatment	Organ	%ID/g ± SEM
Vehicle	Liver	0.47 ± 0.36
	Kidney	1.11 ± 0.47
	Muscle	0.10 ± 0.03
	Tumor	0.86 ± 0.30
	Tumor/Liver	3.09 ± 0.9
	Tumor/Muscle	8.75 ± 0.5
UII	Liver	0.37 ± 0.08
	Kidney	0.89 ± 0.11
	Muscle	0.11 ± 0.02
	Tumor	0.65 ± 0.17
	Tumor/Liver	1.69 ± 0.5
	Tumor/Muscle	6.47 ± 1.6
urantide	Liver	0.40 ± 0.05
	Kidney	1.10 ± 0.14
	Muscle	0.12 ± 0.02
	Tumor	1.01 ± 0.32
	Tumor/Liver	1.88 ± 0.3
	Tumor/Muscle	8.51 ± 8.5

Figure S6 Le Joncour et al.
