# Supplementary material for: Targeting the Urotensin II/UT G Protein-Coupled Receptor to Counteract Angiogenesis and Mesenchymal Hypoxia/Necrosis in Glioblastoma
Source: Front Cell Dev Biol. 2021 Apr 14;9:652544. doi: 10.3389/fcell.2021.652544 (PMC8079989; doi:10.3389/fcell.2021.652544)
Supplement: Supplementary file 2 [file Data_Sheet_2.pdf]

Supplementary Material

1 Supplementary Data

1.1. Supplementary methods

Cell growing assay

Cells were seeded at 3 x 10^6 in 75 cm² flasks, harvested with trypsin-EDTA (1%) and centrifuged (5 min, 0.1 g). Cell pellet was resuspended in the appropriate culture media, seeded in 24-well plates (2.5 x 10^5 cells/well) and incubated in absence or the presence of UII (Phoenix Pharmaceuticals, Inc) (10^{-12} to 10^{-7} M). Cell number was determined in each well after 48 hour-incubation, with an electronic cell counter (Z2, Beckman Coulter). The data were represented as the ratio of cell number normalized to control.

Western blot of HIF-1α/HIF-2α

U87 and hCMEC/D3 cells were exposed to normoxia (20%) or hypoxia (1%) conditions during 4 hours in the absence of FBS and in the presence of UII (10^{-7} M) and/or urantide (10^{-6} M). Cell lysates (20 µg total proteins) were prepared in ice-cold Lysis Buffer (20 mM HEPES, 100 mM NaCl, 1 mM EDTA, 10% NP40, 1X protease and/or phosphatase inhibitor cocktails), mixed with Laemmli buffer and loaded onto 10% SDS-PAGE (sodium dodecyl sulfate-polyacrylamide gel electrophoresis) gels. Proteins were transferred onto a nitrocellulose membrane. Membranes were blocked with 2% nonfat milk or 4% bovine serum albumin, incubated with the primary HIF-1α (Novus, NB100-479), HIF-2α (Abcam, 199-100, Paris, France) or actin antibody (Abcam, 1801) overnight at 4 °C and then with horseradish peroxidase-conjugated secondary antibodies (Santa-Cruz) for 2 h at room temperature. Immunoreactive bands were visualized by using the ECL Western blotting substrate (Promega, Charbonnières-les-bains, France) and their molecular weight were determined by using PageRuler Plus prestained protein ladder (10-250 kDa) markers (Fisher scientific).

Synthesis of the ^{99m}Tc RGD radioligand

Briefly, 7.5–10 mg of HYNICRGD was incubated with 1 ml of an EDDA/tricine mixture (20 mg/mL, 10 mg/ml EDDA, pH adjusted to 8), 1850 MBq of $^{99m}TcO_4^-$ in saline (0.5–1 mL depending on generator calibration) and 20 mg of $SnCl_2$ (20 mL of a tin(II) solution of 1 mg of $SnCl_2$ in 1 mL of 0.1 N aqueous HCl) for 10 min at 100 °C. ITLC strips (dark green) were purchased from Biodex (New York, NY, USA), and the solvents used were methyl ethyl ketone and an acetonitrile/water solution (1:1). A sample of 0.1 mCi of the mixture was submitted to thin layer chromatography (TLC) analysis, and the radiochemical purity was estimated with a MiniGita Star Radiochromatograph (Raytest, Straubenhardt, Germany). Methyl ethyl ketone was used as the eluent for the detection of ^{99m}Tc-pertechnetate (R_f=1), and an acetonitrile/water solution was the eluent for the determination of reduced hydrolyzed ^{99m}Tc (^{99m}Tc colloid, R_f=0).

MicroSPECT image analyses

After 2 hour-post [^{99m}Tc]HYNIC-RGD injection, SPECT scans were acquired using 64 projections over 360° (ROR=9.3 cm, 15 s/ projection). SPECT projections were reconstructed with the filtered back projections algorithm and Hamming filter (0.6 cut-off frequency). Each reconstructed matrix (80x80 pixels) was composed of 80 transverse images with a voxel size of 2.24x2.24x1.5 mm. Further, after SPECT acquisition, CT scans were acquired at 50 kV (320 mA) with 1024 projections over 360° and an FOV of 91 mm. Each reconstructed matrix (512x512) was composed of 512 transverse images with a cubic voxel edge dimension of 0.15 mm.

Visualization and quantification of radioactivity with a β imager

After intravenous injections, resected consecutive 4 mm thick slices were stained with H&E and prepared for β imaging. H&E histological sections were examined under an Olympus DX51 microscope (Olympus, Paris, France). Quantification of necrotic index was performed with Image J software. Quantification of [^{99m}Tc]HYNIC-RGD binding was performed on a high speed Autoradiography β imager (BioSpace Lab, Nesles la Vallée, France). Briefly, dried consecutive sections of tumors were placed in a sample holder inside the detection chamber of the β imager. The levels of bound activity in the tumor sections were directly determined by counting the number of β-particles emerging from the tissue sections. The M3 Vision program (BioSpace Lab) was used to measure the activities in the region of interest (*i.e.* whole tumor section or by discriminating tumor parenchyma of necrotic areas). The radioligand binding signal was expressed in counts per minute per square millimeter (cpm/mm²).

Data analysis

Reconstructed data from SPECT, CT and planar imaging were analyzed using OsiriX-64 imaging software (Pixmeo, Geneva, Switzerland). Quantifications of [^{99m}Tc]-HYNIC-RGD uptake in tumors were performed using MATLAB software (MathWorks, Meudon, France), expressed in terms of standardized uptake values (SUVs) and tumor to muscle (T/M) ratios. The T/M ratio was defined as follows. To define the tumor region of interest (ROI), we used a volumetric ROI that encompassed the entire lesion. To define the muscle ROI, a circular ROI (diameter 7 mm) was placed in both limbs; the mean value between these two ROIs was used to compute T/M ratios. The SUV_{mean} was computed according to the following formula: SUV = [tumor activity (Bq/mL)]/[injected activity (Bq)/animal weight (g)], assuming a density of 1 g/cm^3. The SUV_{max} was defined as the maximum voxel value.

2. Supplementary Figures and Tables

2.1. Supplementary Table

60°C/20s, a step specific for UII mRNA (73°C/5s), and 95°C/1s using the primers specified in Table S1. Minus-reverse transcription ("-RT") controls were systematically performed and quality of PCR products was evaluated by generating a melting curve, which was also used to verify the absence of PCR artifacts (primer dimers) or non-specific PCR products. Samples were amplified at least in triplicates (3 different culture flasks and 3 different RT) and relative mRNA copy levels were determined using the comparative ΔΔCt method. Glyceraldehyde-3-phosphate dehydrogenase (GAPDH) or Ubiquitin C (UBC) transcript levels were used as a reference, to control mRNA levels and stability within each cell line. Results were analyzed by using the Quantstudio design and analysis software (Applied Biosystems) and are expressed as mean of gene of interest expression relative to GAPDH or UBC reference gene.

2.2 Statistical analyses

Data were expressed as mean ± SEM and GraphPad Prism (version 5; GraphPad Software, Inc, La Jolla, USA) was used for statistical analyses. Student t test was used for parametric comparisons between paired variables, the Mann-Whitney U test was used for nonparametric pairwise comparisons, multivariate analyses were done with ANOVA with post hoc Dunnett (in vitro analyses) or Bonferroni tests (e.g., in vivo studies) as appropriate, and survival curves were generated by the Kaplan-Meier method. All reported P values were two-sided and considered to be statistically significant at $P < 0.05$.

Gene	Primers
UTS2R	FW : 5'-CCCAACGCAACCCTCAACA-3'<br>REV : 5'-ACCACGTAGACGTACATGGAG-3'
UTS2	FW : 5'-AGGAAATTTGAGAAAGTTTCAGGAR-3'<br>REV : 5'-ATGGGTGTTTCTGAGCTGACT-3'
UTS2D	FW : 5'-CCCAGCCAACATAATGGAGC-3'<br>REV : 5'-GACTTGGATCCTTCTGCTTCTGT-3'
MMP9	FW : 5'-TGTACCGCTATGGTTACACTCG-3'<br>REV : 5'-GGCAGGGACAGTTGCTTCT-3'
ITGAV	FW : 5'-TCGGATCAAGTGGCAGAAATC-3'<br>REV :5'-AAATCTCCGACAGCCACAG-3'
GAPDH	FW : 5'-CTGGGCTACACTGAGCACC-3'<br>REV :5'-AAGTGGTCGTTGAGGGCAATG-3'
UBC	FW : 5'-CTGGAAGATGGTCGTACCCTG-3'<br>REV :5'-GGTCTTGCCAGTGAGTGTCT-3'

Supplementary Table S1. The primer sequences used for amplification of human UTS2, UTS2D, UTS2R, MMP9, ITGAV mRNAs and the mRNA encoding the housekeeping gene GAPDH or UBC. UBC, Ubiquitin C gene, GAPDH, Glyceraldehyde 3-Phosphate DeHydrogenase.

2.3. Supplementary Figures Legends

Supplementary Figure S1. UII-related migratory effects in U87, hCMEC/D3 and HUV-EC-C cells. A, Quantification of U87 (**A**), hCMEC/D3 (**B**) and HUV-EC-C (**C**) cell number in the absence (control) or the presence of FBS 10% or UII (10^{-12}, 10^{-11}, 10^{-10}, 10^{-9} or 10^{-8} M) after 24h treatments. Statistical significance of treatments *vs* control condition was assessed with one-way ANOVA with Dunnett *post hoc* test. *, $P<0.05$; **, $P<0.01$; ***, $P<0.001$.

Supplementary Figure S2. Effects of the urotensinergic ligands on weight loss and toxicity in *Nude* mice xenografted with U87-MG cells. During the *in vivo* tumor growth experiment (Figure 4), *Nude* mice were treated daily with intra-tumoral injections of UII (0.29 ng/kg, n = 10), palosuran (29 ng/kg), UII+palosuran (0.29/29 ng/kg, respectively, n=10), urantide (29 ng/kg, n=10), UII+urantide (0.29/29 ng/kg, respectively, n=10) or vehicle (PBS, 10 µL, n = 10). Mice weight was controlled every two days and no apparent toxicity of the molecule was seen.

Supplementary Figure S3. Effects of a high dose of urantide on tumor growth. A, Tumor growth of *Swiss Nude* mice transplanted with U87 glioblastoma treated as in Figure 5D with vehicle (10 µL, n=10), UII (0.29 ng/kg, n=10) or urantide (290 ng/kg, n=10). High dose of urantide induced a persistent inhibition of tumor growth. Statistical significance for tumor growth was given by using two-way ANOVA with Bonferonni *post hoc* test compared with vehicle. **, $P < 0.01$; ***, $P < 0.001$. **B**, Late tumor growth of *Swiss Nude* mice transplanted with U87 glioblastoma treated with vehicle (10 µL, n=7) or urantide (29 ng/kg, ↓, 290 ng/kg, ↑, n=10) when tumor volume reached 500 mm^3. The higher dose of urantide induced inflexion of the tumor growth kinetic for one week duration. Statistical significance for tumor growth was given by using two-way ANOVA with Bonferonni *post hoc* test compared with vehicle.

Supplementary Figure S4. Western blot of U87 (A) and hCMEC/D3 (B) cells exposed to normoxia or hypoxia and expression of HIF-1α or HIF-2α. U87 (**A**) and hCMEC/D3 (**B**) were incubated under normoxia (20% O_2) or hypoxia (1% O_2) during 4 hours in the absence of FBS or in the presence of UII (10^{-7} M) and/or urantide (10^{-6} M). β-actin antibody was used as protein loading control.

Supplementary Figure S5. Impact of UII on MMP9 and ITGAV alphav integrin gene expression in GBM and hCMEC/D3 cells. Cells were treated with UII (10^{-9}M, 24 h) in the absence of FBS, and mRNAs were extracted for RT-qPCR analysis. MMP9 and ITGAV mRNA expressions were presented as ΔΔCt, related to the UBC gene expression in GBM and hCMEC/D3 cells. Data were expressed as mean ± SEM of three independent cultures, and normalized to the housekeeping gene UBC. Statistical significance of treatments vs control condition was assessed with Mann-Whitney test. *, $P<0.05$.

Supplementary Figure S6. Schematic representation of *in vivo* protocols used for MicroSPECT imaging and quantification of ^{99m}Tc-RGD. A, schematic protocol description (*left*) and structure of the ^{99m}Tc-RGD tracer (*right*) used to visualize and quantify $\alpha_V\beta_3$ integrins in living xenografted animals. **B**, Brief summary of typical ^{99m}Tc-RGD conducted experiments. **C**, Gamma rays quantifications for selected organs, *i.e.* liver, kidneys, muscle and tumor for animals used in Figure 9.
